# An Integrated Raman Platform with Embedded AI for Intraoperative Real-Time Cancer Detection

**DOI:** 10.1021/acs.analchem.6c00260

**Published:** 2026-07-10

**Authors:** Dinkar Regmi, Ya Zhang, Huaizhi Wang, Zheng Li, Jian Xu

**Affiliations:** Division of Electrical and Computer Engineering, 5779 Louisiana State University, Baton Rouge, Louisiana 70803, United States

## Abstract

Raman spectroscopy workflows are often fragmented across proprietary tools and ad hoc data-processing scripts, which slow decision-making and limit reproducibility and auditability. We present an integrated Raman platform with a Python-based GUI application that interfaces with the optical system(s) to unify instrument control, automate data acquisition, noise removal, signal processing, and run an embedded AI model for real-time classification of experimental samples. The system automatically archives intermediate and final outputs for auditability. We validated the platform’s output for two types of experimental specimens: Tylenol and peritumoral biological samples from human laryngeal tissues. We compared the results with other studies, commercial software, and previous data-processing algorithms. The results obtained were consistent across all comparators. To demonstrate native analytics within the same workflow, we also developed and embedded a 1D convolutional neural network (CNN) tailored for biological Raman spectra. The multilayered CNN was trained on *ex vivo* human laryngeal tissue Raman spectra and was evaluated across 50 independent runs. The model achieved a mean test accuracy of 0.8929 ± 0.0213, a sensitivity of 0.9086 ± 0.0321, a specificity of 0.8698 ± 0.0434, and a mean AUC of 0.9506 ± 0.0142. The average latency across 50 runs was 2.82 s from signal acquisition to prediction, with device acquisition accounting for most of the time. The key innovation is not only the embedded AI but also an end-to-end, auditable workflow that unifies device control, acquisition, signal processing, visualization, and the archival of intermediate and final outputs, with optional real-time inference within a single platform.

## Graphical abstract

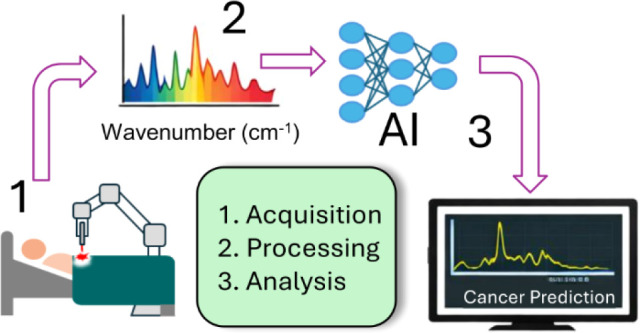

## Introduction

Cancer is one of the leading causes of human deaths in the world. The American Cancer Society estimates 2,042,910 cancer cases and 618,120 cancer deaths in the year 2025, in the US alone.^1^ Accurate diagnosis and early detection play key roles in treatment, saving patients’ lives. To remove solid tumors, during surgery, it is crucial to confirm that all cancerous tissue has been removed with “clear margins”. However, current intraoperative and point-of-care margin assessments are expensive, infrastructure-intensive, and time-consuming, which may ultimately delay decision-making in the operating room and increase the risk of incomplete resections.

To this end, our lab has been conducting innovative research in cancer diagnosis using Raman Spectroscopy and AI (Artificial Intelligence).^2–7^ Raman Spectroscopy is a label-free, minimally invasive optical technique. In brief, in this technique, a high-energy light source, usually a laser of a specific wavelength, is directed at an experimental specimen. When light hits the target molecules, it mostly elastically reflects. However, a tiny fraction, typically less than 1 in 1,000,000 photons, scatters inelastically.^8–10^ Depending on the interaction between the molecule and the light, the light either loses or gains energy in minuscule amounts, slightly shifting the incident light’s wavelength. Observing these slight changes in wavelength, which is the primary principle of Raman spectroscopy, provides a “fingerprint region” of the chemicals/molecules in the experimental specimen. This data is usually interpreted as plots of Raman shift (wavenumber) vs intensity or counts. Different elements and molecules appear as distinct peaks in this plot, allowing them to be identified. Our lab has used Raman Spectra to train AI models to detect various cancers, including breast,^4^ laryngeal,^3^ and pancreatic^2^ cancers.

Thus, far, we have relied on commercial software and MATLAB (MathWorks Inc., Natick, MA, USA) for most of our data processing needs. In fact, workflow fragmentation is a persistent barrier to the broader deployment of any custom-developed Raman system.^11–14^ Workflow fragmentation, here, points to the use of proprietary software for data acquisition, preprocessing in separate toolboxes (e.g., MATLAB, Python), and offline AI inference. This fragmentation not only raises costs but also limits the technology’s reproducibility, complicates quality control, introduces operator-to-operator variability during data collection and processing, and hinders its point-of-care use, particularly in resource-constrained settings. While several software tools have been reported, there remains a need for a system that integrates instrument control, acquisition support, live data visualization, auditable data pre- and postprocessing, and embedded AI inference into a single streamlined workflow. To address this gap, herein, we have developed an integrated end-to-end Raman platform that unifies hardware connection/control, live spectral visualization, signal acquisition, standardized and auditable preprocessing, and optional embedded AI inference within a single Python-based Graphical User Interface (GUI) application. The software is designed to minimize manual intervention through a consistent workflow that includes steps such as data acquisition, background capture and removal, baseline estimation and removal, smoothing, and normalization, while concurrently preserving intermediate artifacts for auditability. This automation reduces user variability, minimizes operator-dependent errors, lowers the learning curve for nonexpert users, improves throughput, and standardizes sampling and processing conditions, which are key requirements for scalable clinical translation. Here, automation refers to the standardized execution of data processing steps without manual intervention within a user-optimized process flow, rather than fully unsupervised, dynamic automatic optimization of all processing parameters across all sample types. For this study, the preprocessing settings were mostly fixed for consistency across data samples and were mostly derived and optimized from previous studies and experiments.^2–4,6^ At the same time, the platform still provides user-facing controls to review data and adjust key parameters for different sample types and experimental conditions, whenever needed. Implementing the software in Python adds value by providing a modular environment where device communication, signal visualization, preprocessing, data capture, and export can be executed within a single reproducible workflow. Additionally, it also makes the development and embedding of AI models easier. We compared our system’s results with those of commercial software and with our prior MATLAB workflows to evaluate accuracy and reliability. We also developed RamanNet, a multilayered 1D CNN that builds on our previous work^3^ and is specifically designed for biological Raman spectra. The model runs seamlessly within the unified workflow and was trained and evaluated on a larger data set of human laryngeal Raman spectra. We rigorously evaluated the model (RamanNet) across 50 independent runs and report both the best-case and average performance ± standard deviation to quantify stability and mitigate “lucky split” effects. We also calculate and present the platforms’ latencies during several steps in the workflow. Thus, this work, with its integrated workflow (capture → process → predict) and embedded deep learning model, advances Raman spectroscopy toward reproducible and practical deployment for real-time margin assessment and related clinical/research applications.

## Methods

### Raman Spectroscopy System

Our Raman System (Figure 1 and S1) was a custom-made, portable setup comprising a 785 nm diode laser (Turnkey Raman Lasers-785 Series, Ocean Optics Inc.) and a QE Pro spectrometer (Ocean Optics Inc.) with a charge-coupled device (CCD). A Raman endoscopic probe (EmVision LLC.) was used for the light/energy pathway. The bifurcated ends of this optical fiber were connected to the laser source and the spectrometer, while the single end (Raman probe) directed the excitation light to the experimental sample, collected Raman scattering signals, and returned them to the spectrometer. A 3D-printed enclosure (Figure S2) was also used to create an ambient-light-deprived environment during data collection for nonbiological samples. For biological samples, the data were collected in dark lab rooms.

**Figure 1:**
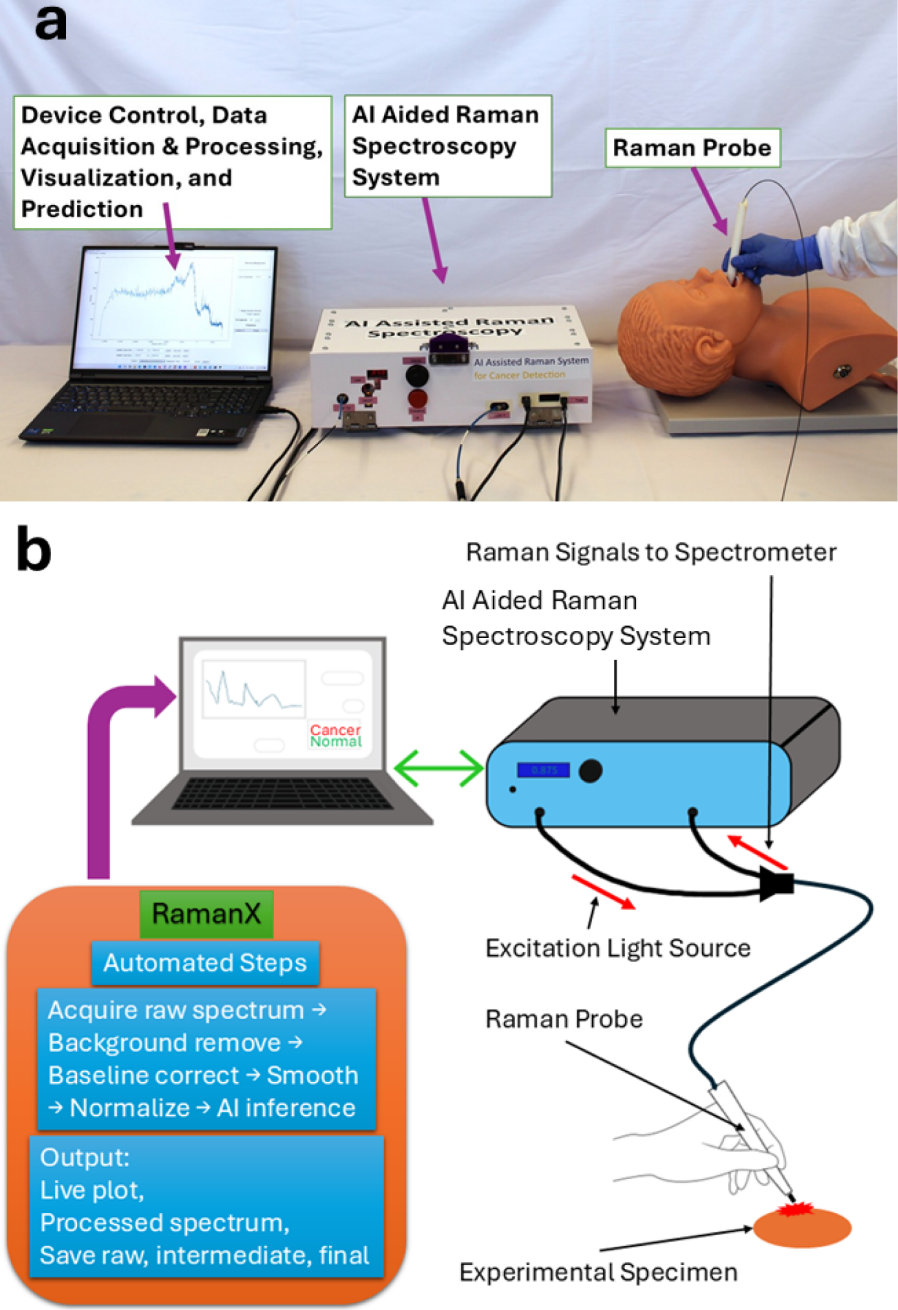
a) Representation of the experimental setup in the real world, b) Schematic representation of the experimental setup using our integrated Raman platform and software (RamanX).

### Data Processing and Software

All data processing and model training, including the graphical user interface (GUI), were implemented in Python unless noted otherwise. OceanView software (Ocean Optics Inc.) and MATLAB scripts from previous studies^2–4^ were used for validation and cross-comparison purposes. The transition to Python was made not only to enable the development and deployment of an AI model but also to consolidate device control, data acquisition, visualization, preprocessing, and archival within a single, maintainable codebase. This reduces repeated data handoffs between commercial and proprietary software, as well as between segregated analysis tools and scripts, and improves data integrity, auditability, and scalability. Equation 1 ^15–17^ was used to convert the raw wavelengths and intensities acquired from the spectrometer into Raman Shift data. In this equation, λ_Reference_ is the excitation wavelength in nanometers (nm), and λ_Scattered_ is the observed wavelength in nanometers (nm). Preprocessing steps included background removal, autofluorescence correction using asymmetric truncated quadratic processing,^2,18^ noise removal and smoothing via Savitzky–Golay filtering,^2,19^ and full spectrum min-max normalization using eq 2. In eq 2, I_Norm_ is the normalized spectrum, I is the acquired spectrum, and *I*_min_ and *I*_max_ are the minimum and maximum intensities, respectively, within the same spectrum. Due to the inherent nature of how SciPy’s Savitzky-Golay function works in Python, the window length was set to be 11 (versus 10 earlier in MATLAB), and the polynomial order was maintained at 2. Despite this mandated discrepancy, we will show that the results are mostly identical between the two systems. For the experiments reported in this study, these preprocessing settings were applied in a fixed and standardized manner to ensure consistency of evaluation across the data sets. However, it should be noted that the software is not restricted to rigid and fixed preprocessing parameters. It includes user-facing modules for selecting different smoothing functions, cost functions, and their associated parameters. Users can review and modify these variables in real time and make adjustments as required by the experimental needs and the spectra type. Python module Seabreeze^20^ was used for device control and data acquisition. SciPy,^21^ NumPy,^22^ and pandas^23,24^ were used for data processing and manipulation. Matplotlib^25^ was used for visualization of plots and graphs, and Tkinter (a Python native module) was used for GUI interface design. An integration time of 3 s was used to capture all spectra. Further, the Seatease module^26^ was used in the software to provide additional functionality for simulating a virtual spectrometer (Figure S3). To reduce the risk of overprocessing or malfunctioning, intermediate outputs can be visualized during the workflow and preserved for later review. This allows users to verify that diagnostically relevant peaks are retained after background subtraction, baseline removal, smoothing, and normalization.

1 $$Raman\;shift\left(cm^{-1}\right)=\left(\frac{1}{\lambda_{reference}}-\frac{1}{\lambda_{scattered}}\right)\times 10^{7}$$

2 $$I_{Norm}=\frac{I-I_{min}}{I_{max}-I_{min}}$$

### Non-Biological Experimental Sample

For calibration and validation purposes, nonbiological reference specimens, generic-brand acetaminophen (Tylenol) tablets purchased over the counter, were analyzed. This specimen was chosen for its wide acceptance as a calibration and validation specimen and for its ease of availability and nontoxicity compared to other widely used specimens, such as cyclohexane and Toluene. The tablets were used as purchased without modification and measured in a custom 3D-printed enclosure (Figure S2) to minimize ambient light interference during data acquisition.

### Biological Experimental Sample

Raman spectra for human laryngeal tissues were collected as described in our previous study.^3^ Briefly, human laryngeal cancer specimens were collected from patients undergoing surgical resection at Our Lady of the Lake Hospital, Baton Rouge, Louisiana, USA. Informed consent was obtained from all subjects, and all procedures were conducted under IRB approval (IRB #4267) in LSU A&M (Louisiana State University). The data set used in this study includes a significantly larger number of specimens than in our prior work, thereby improving statistical robustness. A combination of total laryngectomy and transoral microsurgical laser resection cases was included. All specimens were oriented and labeled according to standard surgical pathology protocols, and Raman spectra were collected from both tumor core regions and the labeled surgical margins. Data from 23 patients were analyzed.

### Data Set for AI Model

Raman spectra were acquired *ex vivo* from both tumor tissues and adjacent normal margins at approximately 2 mm probe distance using 130 mW laser power and a 3-s exposure time per scan. A total of over 2,212 spectra were collected, with each specimen contributing multiple scans from different regions. 1320 spectra were from cancer specimens, and 892 spectra were from normal margins. The spectral range was 508–3972 cm^–1^ with a resolution of 7–8 cm^–1^.

### CNN (AI Model)

We developed a custom one-dimensional convolutional neural network (1D-CNN), referred to as RamanNet, using PyTorch^27^ for the binary classification of Raman spectra as cancerous or noncancerous. The model’s architecture was designed for biological Raman spectra, where diagnostically relevant information is distributed along a one-dimensional spectral (wavenumber) axis as local peak shapes, relative peak intensities, neighboring band relationships, and broader biochemical band patterns. The earlier layers used relatively larger kernels to capture broader spectral structures and local peak neighborhoods, while the deeper layers used progressively smaller kernels to capture narrower discriminative features. Padding was used to preserve the positional information on spectral peaks during hierarchical feature extraction. Each convolution layer was followed by batch normalization and the ReLU (Rectified Linear Unit) activation function to stabilize training, and dropout layers were interleaved to reduce overfitting in the setting of noisy biological spectra and a limited patient cohort. After feature extraction, a global average pooling layer was used to aggregate distributed features across the spectrum before the final fully connected classification layer. Thus, the benefit of the deeper architecture in this study is not simply an increase in parameter count, but its ability to learn multiscale spectral representations directly from biological Raman data. RamanNet (Figure 2) consisted of seven 1D convolutional layers, each followed by batch normalization and the ReLU (Rectified Linear Unit) activation function. Three dropout layers, the first two with 20% and the last with 50%, were interleaved to reduce overfitting. The convolutional blocks successively reduced the channel depth from 440 to 8, with kernel sizes decreasing from 15 to 3, and padding configured to maintain spatial resolution. After feature extraction, a global average pooling layer was applied to aggregate features across the entire spectral dimension. The resulting feature vector was passed to a fully connected layer with a single output for binary classification. The model used the binary cross-entropy with logits (BCEWithLogitsLoss) and was optimized with the Adam optimizer at a learning rate of 1e^–5^ and weight decay of 1e^–5^. The input data set was split into 80% for training, 10% for validation, and 10% for testing using stratified random sampling. Data were loaded using PyTorch’s DataLoader with a batch size of 32. Model performance was evaluated using accuracy, sensitivity, and specificity metrics on the test set.

**Figure 2:**
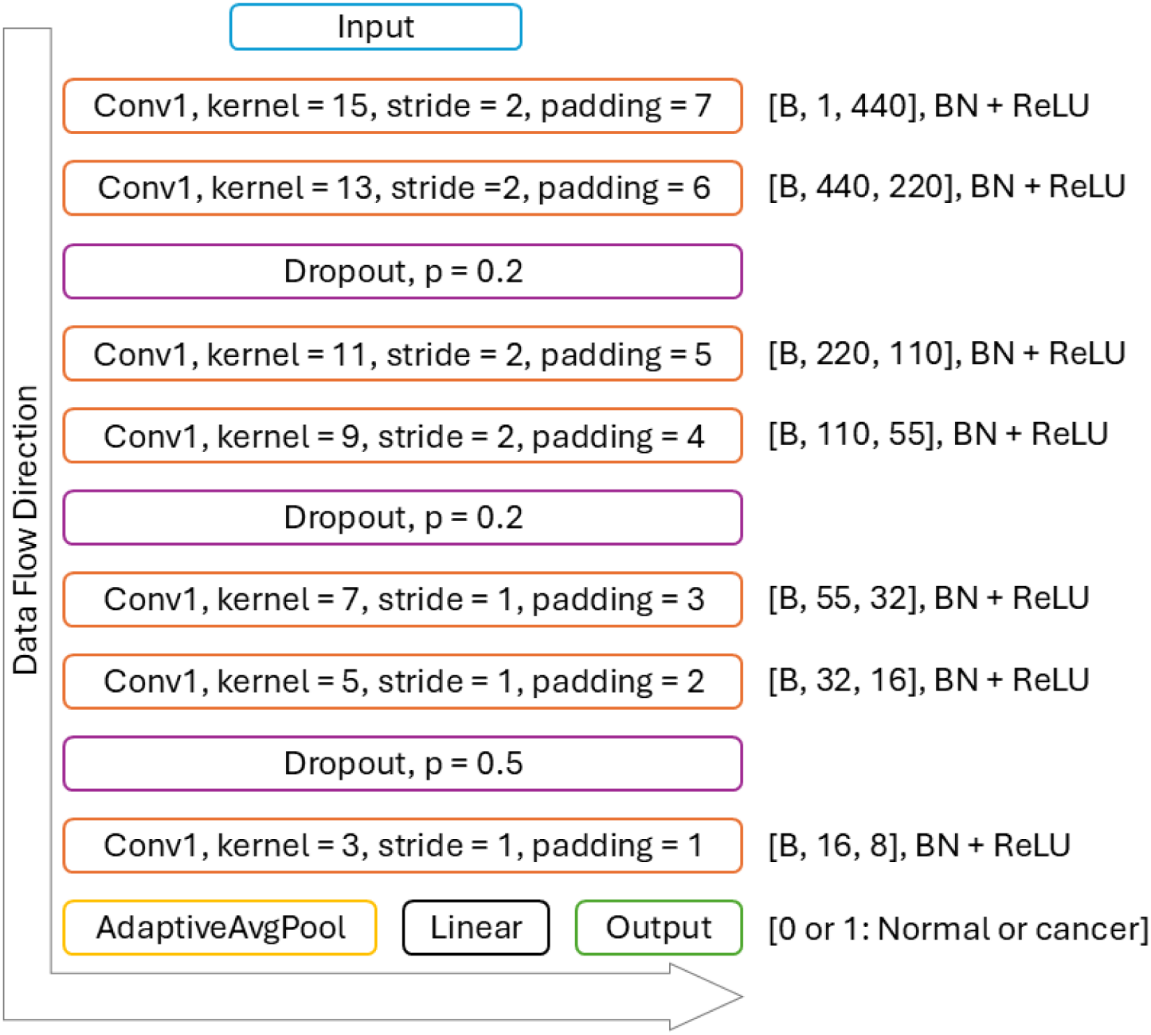
Schematic representation of the 1D CNN model (RamanNet) developed in this study. BN = Batch Normalization, RELU = ReLU activation, Conv1 = Conv1D.

## Results and Discussion

### Overview of the Software (GUI)

Figure 3 shows the front screen of the developed software (RamanX). The top-left part of the GUI displays the Raman spectra. The bottom-left section contains the functions for plot updating, device management, and data acquisition. This section also displays the integration time and includes the data capture button. The top-right portion contains the background removal function, followed by a display of the laser’s wavelength (in nanometers, nm) and a function to update this reference wavelength, which is used in eq 1 to calculate the Raman shift. This same update function also allows for updating the integration time in a separate window (Figure S4). The right bottom section of the window shows the number of data samples (when) captured, has a button that allows the user to save collected/captured data, and a check button to allow the software to predict, using a pretrained/preselected AI model (Figure S5), whether the data being captured shows cancerous traits. The prediction for each data sample capture is shown in the bottom-right-most portion of the window under the section called “Predictions”. Additionally, the software offers more auxiliary windows for users to review and adjust key processing operations. For example, the filter view window (Figure S6) allows the user to choose an appropriate smoothing filter and size. Baseline view window (Figure S7) allows users to inspect the estimated baseline and adjust related variable and cost functions. Similarly, the smooth and normalized view window (Figure S8) allows users to examine the normalized output before they decide to proceed forward in the workflow. A video tutorial demonstrating the real-time use of RamanX, including device connection, acquisition, preprocessing review, saving, and optional prediction, is provided in the Supporting Information.

**Figure 3:**
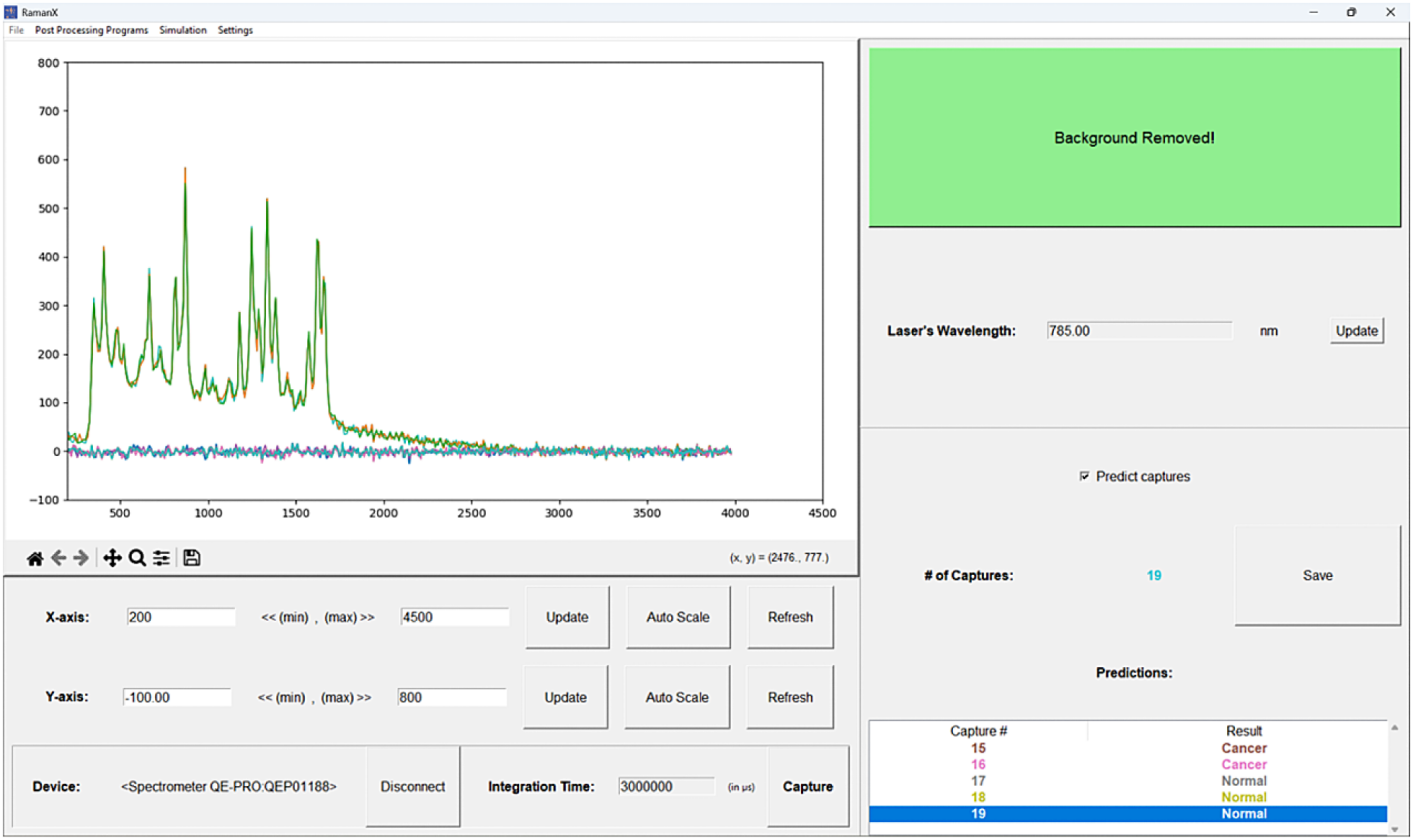
Front screen of the developed software (RamanX). Displayed here for illustrative purposes only. The Raman spectra shown correspond to Tylenol.

### Overview and Results of the Data Processing Methods

Figure 4 diagrammatically illustrates the software’s workflow. First, once the hardware device is set up and ready to collect data, the software automatically scans for connected devices and allows users to connect to any available device (Figure S9). Once connected, it acquires raw wavelength and intensity data from the spectrometer and converts them into a Raman spectrum using eq 1. In the next step, a background signal is captured and saved in the memory. To do so, the system’s workflow protocol dictates that the Raman probe be pointed at the experimental specimen and that data be collected in a light-deprived environment (Figure S10). The captured background signal is subtracted from all raw data from this point forward. The laser source can then be turned on, and the acquired Raman spectrum is displayed in the software. Here (at Step 3 in Figure 4), the software allows to start the data capture process. For each spectrum captured, an overlay plot is displayed in the plot area, and a capture count number is rerecorded. Both plots and counts are color-coded for easy identification. If, in addition, real-time predictions are desired for the captured Raman spectra, the software proceeds to steps 4 onward in Figure 4. In such cases, the background-subtracted data from Step 3 is passed through the asymmetric truncated quadratic function to calculate a baseline, which is then subtracted from the previous data in Step 4 (Figure S7). The resulting data is then smoothened using Savitzky–Golay filtering, followed by full-spectrum min-max normalization (eq 2). At this point, the data becomes ready to be fed into the desired AI models. The prediction results are then shown on the screen in a color-coded format with the appropriate capture number. From Step 3 onward, the captured data can be saved in Microsoft (MS) Excel files. In fact, whether prediction is desired or not, whenever possible, the software always saves two data files: one from Step 3 and the normalized version from Step 5. Although these processing steps are executed automatically in this workflow, they are not intended to be a blind transformation or rigid and fixed. The software allows users to inspect the baseline fit, choose appropriate filters and parameters, and preserves the intermediate outputs so that each processing stage can be visually verified and adjusted before the final normalized spectrum is used for AI inference. For example, in cases of a strong fluorescence background, this visual inspection becomes important. In such cases, weak Raman features could be mistaken as part of the baseline and discarded. Currently, the software provides visual confirmation and parameter adjustments. The software design helps address edge cases by preserving both preinference and postprocessing spectra for auditability and comparison, rather than relying solely on the final processed output. Furthermore, this unified execution removes the need to manually transfer data between commercial acquisition software, standalone preprocessing scripts and tools, and separate inference pipelines. In practice, this reduces version mismatch, manual handling errors, and operator-to-operator variability while enhancing reproducibility and auditability.

**Figure 4:**
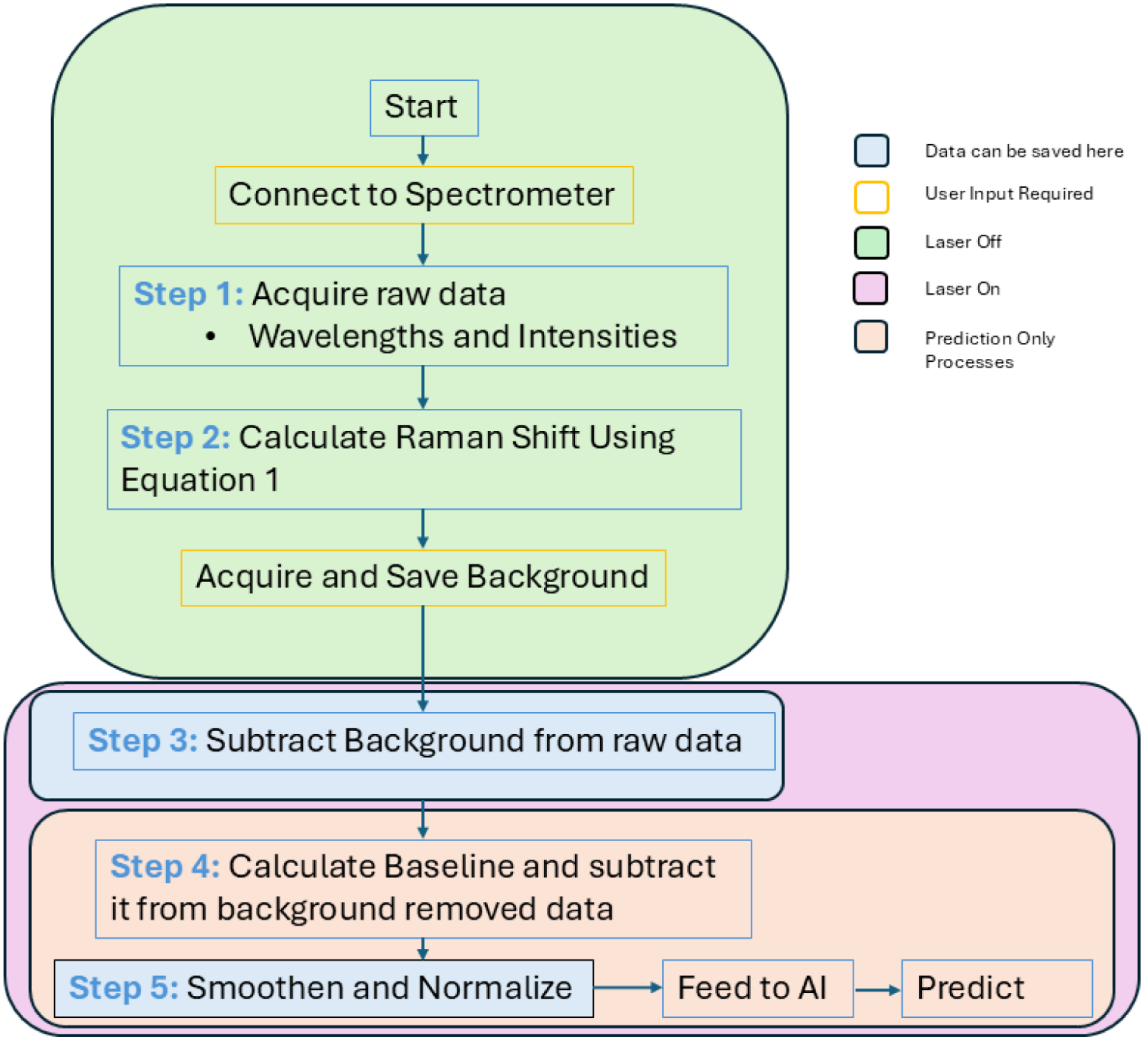
Overview of the software (RamanX) workflow that standardizes Raman signal acquisition, processing, and analysis (by embedded AI).

Figure 5 is included to supplement Figure 4 in illustrating the software’s data processing steps, and to present the final Raman spectra generated for Tylenol with its characteristic peaks. All plots in these figures, except the background signal, show the average Raman spectrum obtained from 10 consecutive Raman spectra captured for Tylenol. Of interest are Figure 5e and f. Figure 5e shows the Raman spectra generated for Tylenol after background removal and baseline subtraction. Figure 5f shows the same plot after Savitzky–Golay filtering and normalization, including some of the prominent peaks observed for Tylenol in this study. These peaks are presented in Table 1. The results shown in Figure 5 not only illustrate the workflow but, combined with Table 1 and the following discussion, also demonstrate that key spectral features persist even after all the employed data processing steps, i.e., background subtraction, baseline removal, smoothing, and normalization.

**Figure 5:**
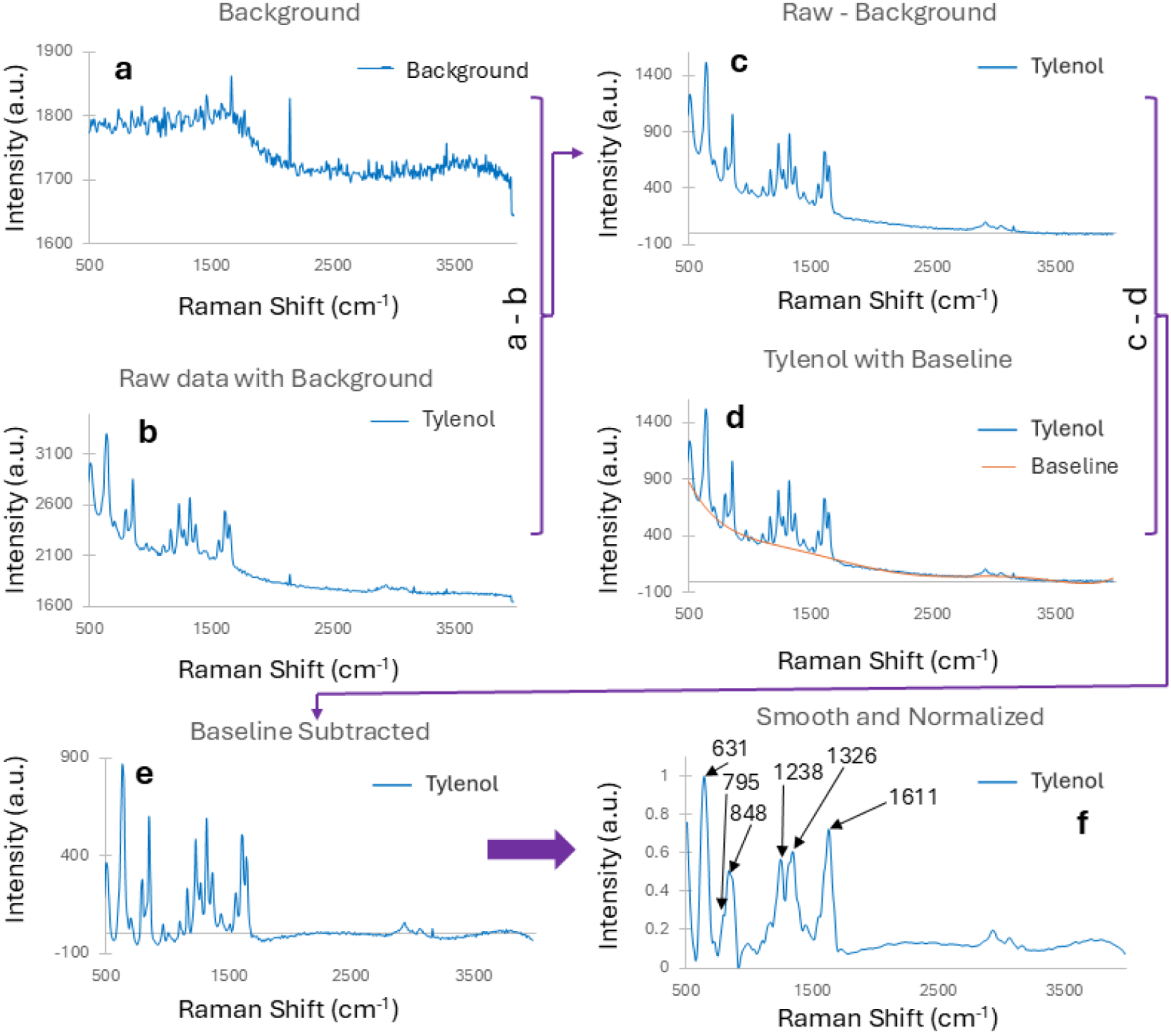
Illustration of the software workflow and data processing using Tylenol’s Raman spectra. (a) Captured background, (b) Raman spectrum (raw data) with the background, (c) Raman spectrum of Tylenol after background subtraction, (d) Raman spectrum after Baseline fitting, (e) Raman spectrum after baseline subtraction, (f) Raman spectrum after Savitzky–Golay smoothing and normalization.

**Table 1:** Prominent Raman Peaks Observed for Tylenol and Human Laryngeal Tissue

Sample	Measured (This Work)	Literature Match	Δ (cm^–1^)	Assignment	References
Tylenol	795	792	3	CNC Ring Stretching	^28^
		797	2		^29^
	848	853	5	Ring Breathing	^28^
	1238	1236	2	C–C Ring Stretching	^29^
	1326	1324	2	Amide III	^29^
		1320	6		^28^
	1611	1611	0	Ring Stretching	^29^
Human Laryngeal Tissue	Positive Region: Cancer Peak > Normal Peak				
	1005	1004	1	Amide III, proteins	^30^
				Phenylalanine ring breathing mode	^31^
	1087	1078	9	lipids	^32^
		1077	10	GSH	^3^
	1546	1554	8	Amide III	^32^
		1536	10	Glutathione (GSH)	^3^
	1555	1554	1	Amide III	^32^
	1639	1643	4	Proteins	^30^
		1638	1	GSH	^3^
	1657	1655	2	Amide I	^30,32^
				Protein amide I band (C=O stretching mode of proteins)	^31^
				Amide I, C=O stretching	^3^
	773	773	0	l-Alanine^a^	^33^
	1188	1188	0	15-Methylpalmiticacid (17iso)^a^	^33^
	1307	1307	0	Adenine^a^	^33^
	1855				
	Negative Region: Cancer Peak < Normal Peak				
	719	727	8	deoxyribonucleic acid (DNA)-based adenine, bending	^3^
	1433	1440	7	Lipids	^30^
	686	686	0	Glycine^a^	^33^
	1423	1423	0	L-tryptophan, Malic acid^a^	^33^

a Potential matches for biological specimens.

In this study, Figure 5f shows the final format of the data passed to downstream processes, such as AI training and prediction. Figure 6a shows the average of the normalized Raman spectra of normal and cancerous human laryngeal tissue used in this study to train and evaluate the AI model. Figure 6b shows the difference between the average Raman spectra of cancerous and normal laryngeal tissue, with several prominent peaks observed. These peaks are also highlighted in Figure 6a for reference. Table 1 and the following section discuss these peaks in more detail.

**Figure 6:**
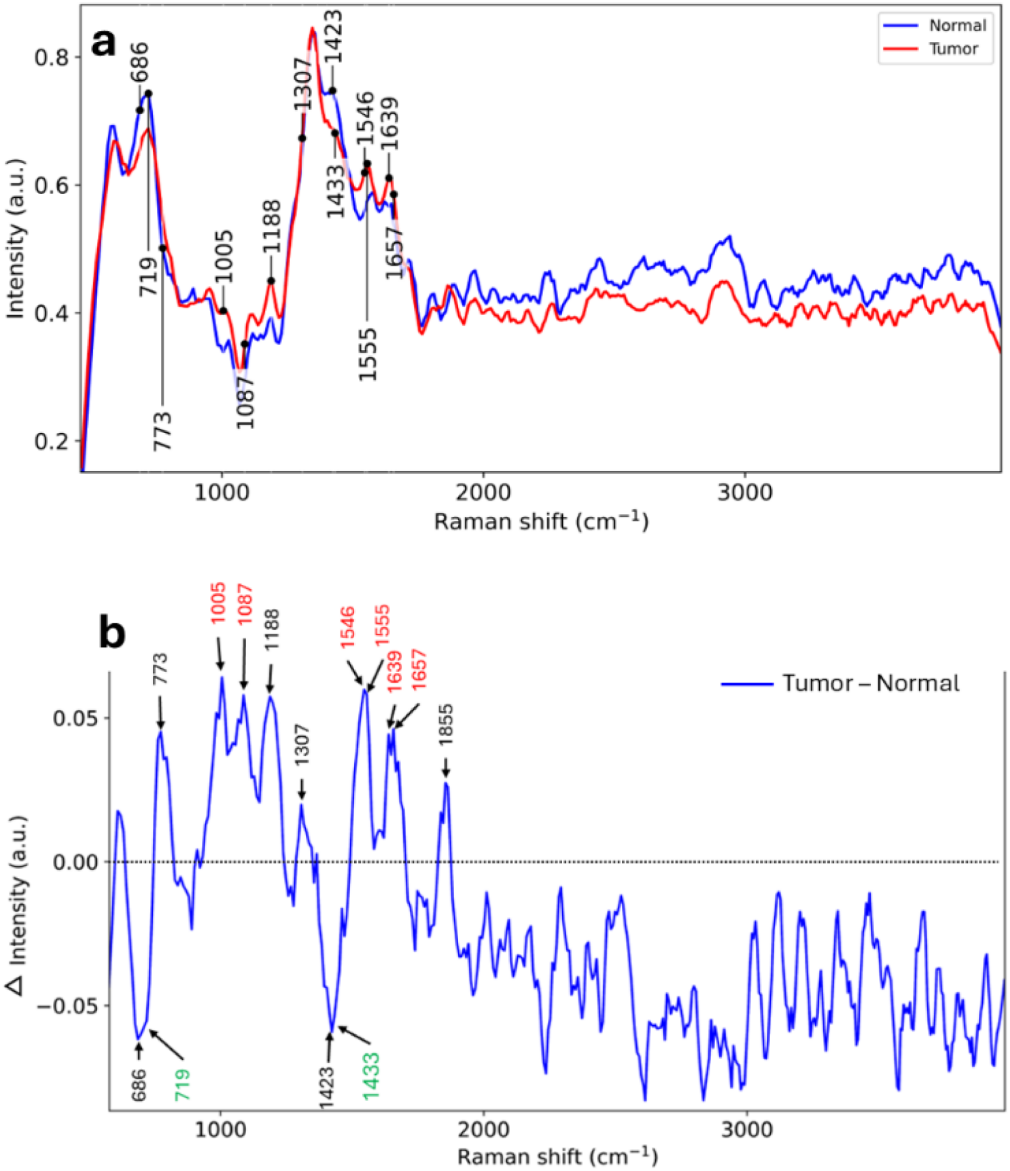
Raman spectral comparison of normal and cancerous human laryngeal tissues. (a) Average normalized Raman spectra, where the blue curve represents normal tissue and the red curve represents tumor tissue; selected prominent Raman bands are labeled. (b) Difference spectrum obtained by subtracting the average normal spectrum from the average tumor spectrum (Tumor – Normal), with labeled peaks indicating regions of relative spectral difference between the two tissue types.

### Validation of the Results

The Raman spectra of Tylenol in Figure 5 show prominent peaks that align closely with those reported in other studies. Peaks at 792, 853, and 1320 cm^–1^ are commonly attributed to Tylenol.^28^ Peaks at 797, 858, 1236, 1324, and 1611 cm^–1^ are usually attributed as canonical bands for APAP (Acetaminophen, also called Paracetamol) and are assigned to CNC ring stretching, ring breathing, C–C ring stretching, amide III, and ring stretching, respectively.^29^ Acetaminophen is an active ingredient in Tylenol (Figure S11). A peak at 631 cm^–1^ was also detected by the system, which is commonly attributed to Titanium Oxide (TiO_2_),^34^ and is, interestingly, also listed as an inactive ingredient in the commercial Tylenol tablets (Figure S11). Similarly, in Figure 6, prominent peaks observed at 1005, 1087, 1546, 1555, 1639, and 1657 cm^–1^ are generally attributed as identifying peaks for cancerous laryngeal tissue.^3,30–32^ These peaks are usually assigned to the phenylalanine ring breathing mode, lipids, Glutathione (GSH), Amide III, proteins, and Amide I, respectively. On the other hand, studies have shown that peaks at 719 and 1433 cm^–1^ in the Raman spectrum are usually larger in normal laryngeal tissue than in cancerous tissue.^3,30^ In addition to these, peaks at 773, 1188, and 1307 cm^–1^ in the positive region, where cancer peaks are higher than normal peaks, and 686 and 1423 cm^–1^ in the negative region, where normal peaks are higher than cancer peaks, were also observed in this data set. Although this study does not perform a direct feature-attribution analysis for RamanNet, the principal spectral differences observed between cancer and normal tissues in Figure 6 and Table 1 are likely to represent informative regions for classification. In particular, the bands around 1005, 1087, 1546–1555, 1639–1657, 719, and 1433 cm^–1^ were among the most visibly discriminative regions in the present data set.

To further verify that the system produces reliable, accurate Raman signals, we compared our results with those from the commercial software (OceanView). To do this, for Tylenol, 10 consecutive Raman spectra were acquired using the same experimental setup in both systems. These data were then processed separately in these different systems: (i) completely by our system (this work), and (ii) data acquisition by commercial software (OceanView) and processing by our old MATLAB algorithms.^2–4^ Figure S12a shows the results for Tylenol, where the blue plot shows the result obtained using our system fully. In contrast, the red plot shows the result obtained by first acquiring data with the commercial software and then processing it with the old MATLAB algorithms. As can be seen, both systems are highly aligned. Similarly, Figure S12b compares our system’s results with MATLAB results for normal and cancerous human laryngeal tissues. It should be noted that all the data in this figure were acquired using commercial software. Then, for data processing, the thus-acquired data was processed separately using the old MATLAB algorithms and the Python algorithms developed in this study. Simply put, the only difference between these pairs of data (in Figure S12b) was whether they were processed using Python-based (this work) or the old MATLAB-based baseline removal, smoothing, and normalization functions. These results, as shown in the figure, are highly aligned. To quantitatively assess the similarity between these results, we computed Pearson’s Correlation Coefficient, RMSE (Root Mean Square Error), Cosine Similarity, and MAE (Mean Absolute Error). The values for these quantities are shown in Table 2. In both cases (Tylenol and human tissue), a high Pearson correlation coefficient (>96%) was observed. Similarly, low RMSE and MAE values of <10% and <8%, respectively, in the overall data, especially given that these were calculated on normalized data, indicate that both systems are highly aligned. These comparisons also serve as a safeguard against unintended distortions introduced by automated preprocessing, including excessive baseline removal. If meaningful Raman bands were being systematically absorbed into the estimated baseline, stronger disagreement with the commercial software-derived and MATLAB-derived spectra, as well as poorer preservation of expected literature peaks, would be expected. The close agreement with the commercial software and prior MATLAB-based workflows, together with preservation of expected Tylenol and biological Raman peaks, indicates that the standardized preprocessing used here did not materially remove diagnostically relevant spectral features in the tested data sets. These results help build confidence in the system’s ability to acquire and process data accurately.

**Table 2:** Quantitative Similarity between This Work and Previous MATLAB-Based Processing Pipelines

Specimen Type	Specimen	Pearson Correlation	RMSE	MAE	Cosine Similarity
Non-Biological	Tylenol	0.99627	0.02038	0.06897	0.99775
Human Laryngeal Tissue	Cancer Tissue	0.97198	0.09653	0.07335	0.99882
	Normal Tissue	0.96342	0.07381	0.05722	0.99867

### AI Inference

Once satisfied with the data processing capabilities, we focused on the AI component. We ran a convergence test on our model, running it 50 times with 200 epochs each. Figure 7 plots the results for the average of each of these runs. Figure 7a, in combination with Figure 7b, shows that convergence is to be expected between 100 and 200 epochs. We tested this observation, and the empirical data showed that the best results were obtained when running the model for 200 epochs. Figure 7c shows the Receiver Operating Characteristic (ROC) curve for the average of the 50 runs. The results showed that the model had an average test accuracy (on never-before-seen test data) of 0.8929, with a standard deviation of ±0.0213. Similarly, the observed average sensitivity, specificity, and AUC (Area Under the Curve) for the model were 0.9086 ± 0.0321, 0.8698 ± 0.0434, and 0.9506 ± 0.0142, respectively (Table S1). However, the best overall performance, in terms of AUC, was observed to have a test accuracy of 92.79%, sensitivity of 92.42%, specificity of 93.33%, and AUC of 0.9784 (Table S1).

**Figure 7:**
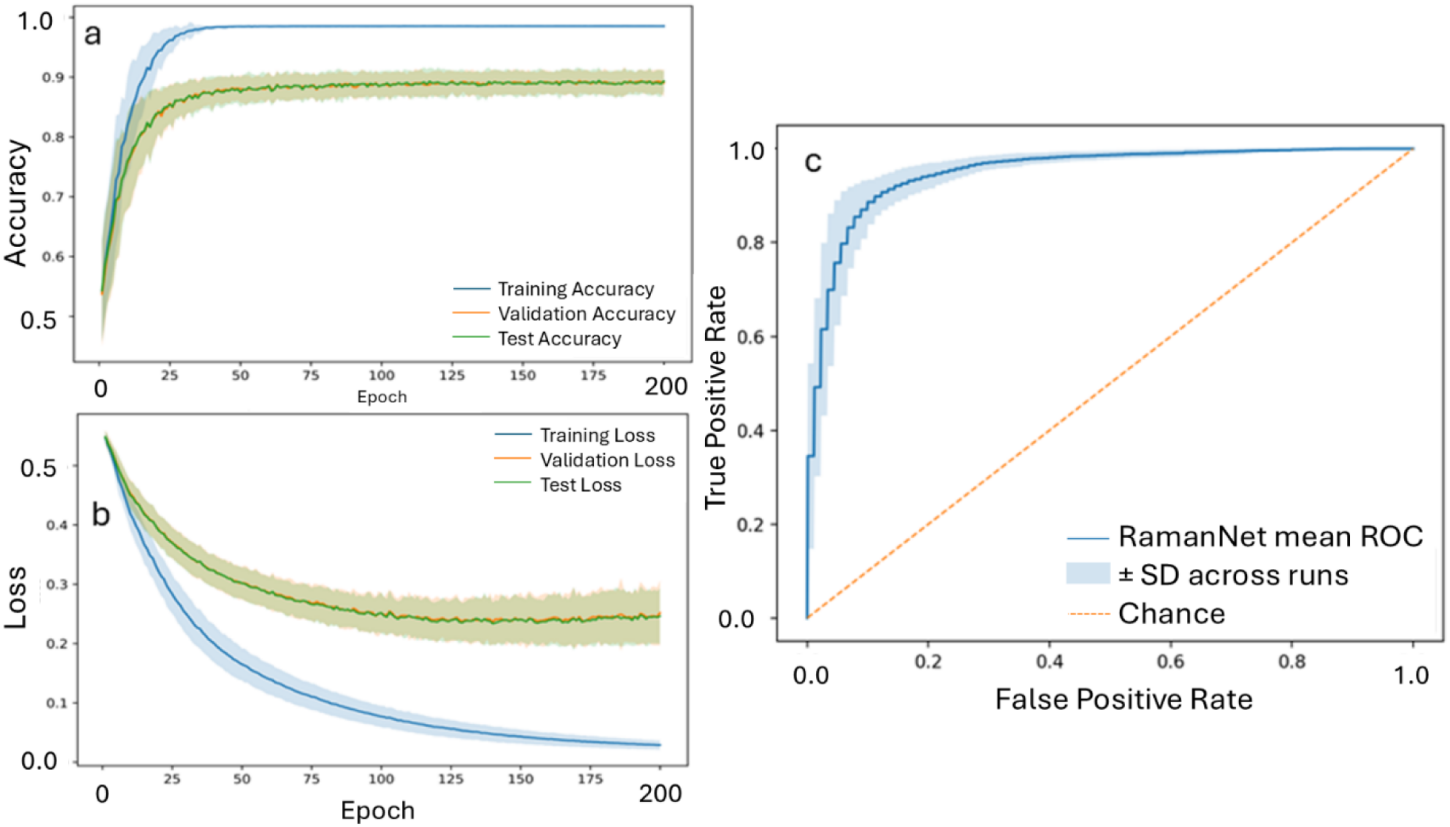
Training performance of RamanNet across 50 independent runs. (a) Mean accuracy ± standard deviation for the training, validation, and test sets over 200 epochs. (b) Mean loss ± standard deviation for the training, validation, and test sets over 200 epochs. (c) Mean receiver operating characteristic (ROC) curve on the test sets across the 50 runs, with the shaded region indicating ±1 standard deviation. The model achieved a mean AUC of 0.951 ± 0.014 for the classification of human laryngeal tissue Raman spectra.

From literature review, the only other study that uses neural networks for classification of human laryngeal tissue into cancerous and noncancerous types was done by Li et al.^3^ The accuracy they achieved was reported to be 96.1% with a sensitivity, specificity, and AUC of 95.2%, 96.9% and 0.98, respectively. However, their patient cohort was much smaller, with only 10 patients, 500 tumor spectra, and 207 normal spectra, and the CNN they used was shallow with just one layer. To compare our model’s performance with theirs, we recreated their model (assuming a ReLU function, since it was not mentioned in the study) and ran it alongside ours at each epoch and run. The results show that on our expanded data set, the model achieved average test accuracy, sensitivity, specificity, and AUC of 87.62%, 89.77%, 84.47%, and ∼0.95, respectively. All these values are lower than those observed for our model. Table S1 compares our approach with other similar studies. Stone et al. collected Raman spectra from 15 human laryngeal tissues and used linear discriminant analysis to achieve an average accuracy of 87.2% across normal and cancer tissues.^35^ Lau et al. used Principal Component Analysis (PCA) in combination with other statistical methods (ANOVA, etc.) to classify cancerous and normal tissues from 21 human subjects and achieved a sensitivity of 79% and specificity of 90.15%.^36^ Teh et al. studied 16 patients and used a Random Forest (RF) model, achieving an accuracy of 89.3%.^37^ Pujary et al. also performed an exploratory study in 20 patients using PCA-based methods.^30^ Lin et al. performed a similar study with a larger cohort of 39 patients, but a smaller number of spectra of 94, using PCA-LDA (Linear Discriminant Analysis) as a classifier and Leave-one-subject-out cross-validation, and achieved an accuracy of 90.4%, sensitivity of 90.3%, specificity of 90.9%, and AUC of 0.97.^38^ In 2016, Lin et al. used Raman Spectra in the fingerprint (FP) region and high wavenumber (HW) region from laryngeal tissues to achieve a test accuracy of 91.1% and an AUC of ∼0.97 using PLS-DA (Partial Least Squares – Discriminant Analysis), a PCA-based technique, as the classification model.^32^ van Lanschot et al. performed a PCA-LDA on 22 human specimens, achieving an AUC of 0.87.^39^

To summarize, most earlier laryngeal Raman studies are either PCA-based (PCA-LDA, PLS-DA, ANOVA/PCA, etc.) or use conventional Machine Learning (ML) models such as random forests (RF). The only other study that used a more advanced neural network model was Li et al.,^3^ albeit with a shallow depth and a smaller cohort. To further benchmark RamanNet against commonly studied machine learning approaches, we evaluated several conventional ML models on the same data set used by RamanNet. The corresponding results are presented in Figure 8 and Table S2. In Figure 8, we compared our model with some commonly used machine learning models, and the single-layered CNN model proposed by Li et al., using four different metrics. In binary classification problems, especially in cancer-related applications, a single evaluation metric may not be enough to show the true diagnostic capability of a model. Therefore, here, we evaluated the performance of all models in terms of accuracy, sensitivity, specificity, and ROC AUC (Area Under the Receiver Operating Characteristic Curve). Accuracy gives a generalized view of the classification capability of a model. Across 50 runs, our model achieved a respectable average accuracy of 89.29%. However, by itself, accuracy is not sufficient because different types of classification errors have different clinical implications. Sensitivity is important because of the risk involved in false negative classification. A false negative classification would mean leaving out the cancerous portion of tissue, which could, in the future, lead to serious consequences such as relapse, resurgery, or loss of life. The average sensitivity observed for our model was 90.86%. Specificity is another important metric because of the nuisances that false positive classifications could cause, such as unnecessary tissue removal, additional sampling, patient burden, and overtreatment. An average specificity of 86.98% was achieved by our model across the 50 independent runs. Another important metric evaluated here is ROC AUC. This metric gives a sense of how well the classifier or model separates cancer from noncancerous spectra across different classification thresholds. A high ROC AUC value suggests that the model is excellent at generally distinguishing cancer spectra from noncancer spectra, even when the decision threshold is changed. Our model achieved an average ROC AUC of 0.9506. We do not propose RamanNet as the better candidate simply because it has more layers, or because it performs the best in every metric all the time. In fact, as can be seen in Figure 8, some simpler models perform better in some metrics. We propose RamanNet as a better candidate because of its balanced and comparable performance across all the diagnostic metrics. Its results are also highly stable, as it was evaluated across many runs, and not just one run. Additionally, it can be easily and directly integrated into the proposed system.

**Figure 8:**
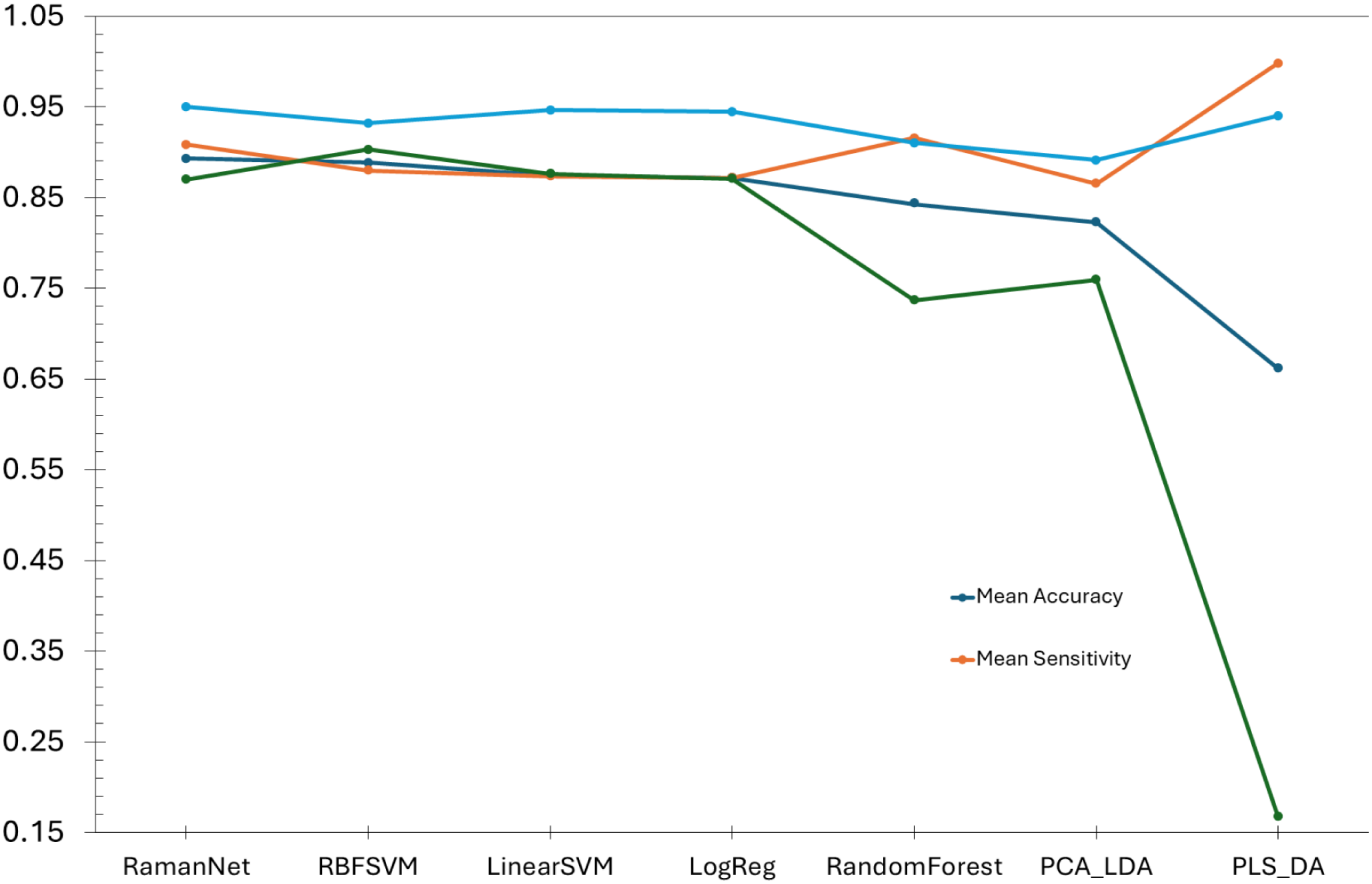
Comparison of RamanNet with other common machine learning models across 50 independent runs using mean accuracy, sensitivity, specificity, and AUC as performance metrics.

To the best of our knowledge, this is the first study to evaluate a deeper 1D CNN model of this type on a human laryngeal Raman data set of this scale (23 patients, 2212 spectra). The motivation behind the model was not depth or an increased number of layers or parameters, but a hierarchical learning structure for biological Raman spectra. RamanNet learns spectral features along the wavenumber axis, with broader filters in early layers to capture broad biochemical bands and local peak neighborhoods, and with smaller kernels in later layers to learn narrower, discriminative spectral patterns. Batch normalization, dropout, and global average pooling layers were integrated into the design to improve robustness against noisy biological Raman spectra. The model was evaluated against a larger data set of human laryngeal tissue Raman spectra than in our previous study.^3^ We do not report a single best run; instead, we ran the model 50 times and report the mean with standard deviation, which shows the model’s stability and reduces “lucky split” ambiguity. The average performance across 50 runs yielded respectable accuracy, sensitivity, specificity, and AUC of 89.29%, 90.86%, 86.98% and 0.9506, respectively. We also compared the model against a recreated shallower CNN baseline and other popular ML models on the same expanded data set, to help separate the architectural benefit from data set size alone. We note, however, that the present work did not include formal CNN explainability analysis, so these spectral interpretations should not be read as direct feature importance outputs from RamanNet itself.

To quantify the real-time performance of RamanX, end-to-end latency was measured across 50 runs by recording timings at multiple workflow stages, including device read-only, preprocessing, inference, click-to-prediction, and overall acquisition-to-prediction. The results are presented in Figure 9. The average overall acquisition-to-prediction latency was 2.82 s, while the average device-read-only latency was 2.75 s and the average click-to-prediction latency was 1.73 s. The lower click-to-prediction latency is due to RamanX’s continuous live acquisition design, which generates predictions from the next available frame after user input rather than from a completely new acquisition initiated at the click. These results show that the dominant contribution to total latency arose from spectral acquisition rather than downstream computation, with preprocessing and inference (0.001645 and 0.001929 s, respectively) contributing comparatively little to the total runtime. This supports the use of RamanX for near-immediate prediction during measurement under the present acquisition settings.

**Figure 9:**
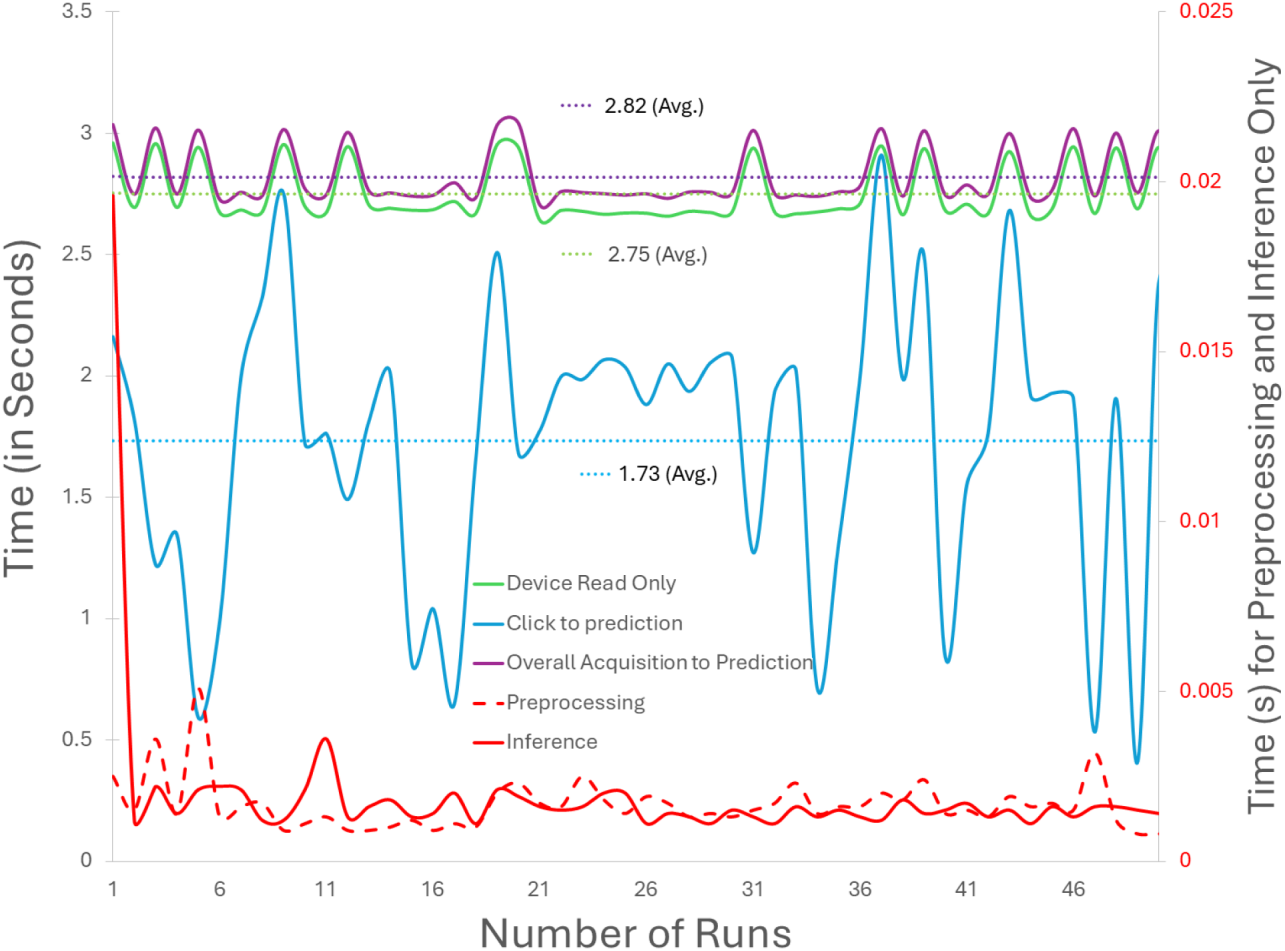
Average latency measured across 50 runs at different stages of the RamanX workflow. Dotted horizontal lines indicate the mean latency for each metric.

Beyond the AI inference and the model itself, the presented implementation consolidates device control, live spectral visualization, auditable data processing, and storage of intermediate and final outputs within a single workflow. Thus, the main advantage of the platform is not only AI embedding but also the consolidation of all these steps, inherent to many Raman data processing frameworks, into a single, reproducible, auditable, and extensible environment, reducing the need to juggle tools, devices, and scripts. This makes the platform feasible for clinical deployment. A literature search reveals that several similar Raman software platforms have been reported. However, most of them focus on limited or selected facets of the system, rather than the whole, as shown in Table 3. For example, Sheehy et al. reported an open-source preprocessing package for Raman signal isolation and baseline handling.^12^ SpectroChat and PyRamanGUI emphasize preprocessing and spectral analysis in GUI form.^40,41^ RamanSPY emphasizes modular Raman data analysis with explicit ML/AI integration.^42^ Earlier systems, such as those by Resner et al. and RAMANMETRIX, include classification or machine-learning workflows.^43,44^ In contrast, AutoOpenRaman and Spectrum Analyzer Suite emphasize acquisition and hardware automation more explicitly.^45,46^ Table 3 presents the capabilities of these systems and their comparison with RamanX. RamanX is positioned not simply as another Raman GUI, but as a platform that combines device connection/control, live spectral display, background handling, baseline removal, smoothing, normalization, saving of intermediate and final outputs, and an option for AI inference, all within a single auditable workflow. This design facilitates research and translational use cases, lowers technical barriers for new users, and provides an auditable, research-grade pipeline for advanced users.

**Table 3:** Comparison of the Current Platform with Previously Reported Similar Software

Platform	Processing/visualization	Real-time acquisition/hardware interaction	Embedded AI in the same workflow	Auditable intermediate outputs	Primary focus
Sheehy et al.^12^	Yes	No	No	Limited	Preprocessing and baseline correction
SpectroChat^41^	Yes	No	No	Limited	Chemometrics analysis GUI
PyRamanGUI^40^	Yes	No	No	Limited	Raman Spectra analysis GUI
RamanSpy^42^	Yes	No	Yes	Limited	Integrative Raman data analysis framework
Reisner et al.^43^	Yes	No	Yes	No	Processing, analysis, classification
RAMANMETRIX^44^	Yes	No	Yes	Yes	Chemometrics, ML Workflow
AutoOpenRaman^45^	Limited	Yes	No	Limited	Automated acquisition, hardware control
Spectrum Analyzer Suite^46^	Limited	Yes	No	Limited	Acquisition, calibration, filtering
This Work (RamanX)	Yes	Yes	Yes	Yes	End-to-end workflow: device control + acquisition + preprocessing + AI inference

### Limitations and Future Work

Although the current platform brings most of the major Raman acquisition and processing steps into a reliable single workflow, several limitations remain. First, the current implementation does not include a fully automatic adaptive parameter optimization algorithm. At this stage, several preprocessing decisions still depend on user judgment, including baseline estimation settings, smoothing filter selection, and related parameter choices. This is particularly important in edge cases, such as severe autofluorescence, which is a common phenomenon in biological Raman spectra, where weak Raman peaks can be partially suppressed by overly aggressive baseline correction. In such cases, the software allows users to inspect the fitted baseline and the processed output, and to adjust the cost function and related parameters in dedicated GUI windows. While this approach helps reduce the risk of overcorrection, a fully adaptive preprocessing algorithm would further strengthen the platform’s ultimate objectives. This remains an important goal for future work.

Similarly, the current system lacks built-in quantitative quality control (QC) metrics for baseline fit quality. At present, preprocessing validity is primarily assessed through visual inspection of intermediate outputs and comparison with expected spectral structure. Furthermore, a separate spike-removal (despiking) step, a common step in Raman preprocessing to handle isolated artifacts such as cosmic-ray spikes, has not yet been implemented. Adding automated quality control metrics, along with dedicated spike detection and removal, would improve the workflow’s robustness and reduce reliance on operator judgment.

Another limitation is that RamanX was developed and validated using the specific hardware configuration as described in this study. It is not yet intended to serve as a universal multidevice Raman control platform. Although the software architecture is modular and adaptation to other devices and probe geometries is feasible in principle, such an extension would require modification to the device communication layer and validation of compatible acquisition settings. Overall, future work will focus on improving the platform’s robustness by incorporating adaptive parameter optimization, quantitative preprocessing quality-control metrics, spike identification and removal, and a more flexible device-integration framework to support broader hardware compatibility.

## Conclusion

This work addresses one of the key barriers to clinical translation of Raman spectroscopy: fragmented workflows that separate acquisition, data processing, and AI inference. We developed and validated an integrated, end-to-end Raman platform that unifies device control, auditable signal processing (background subtraction, baseline removal, smoothing, normalization), and real-time visualization and AI inference in one single Python-based GUI application. The platform’s outputs for reference measurements and peritumoral human laryngeal samples were consistent with the results of the commercial software (OceanView), previously published MATLAB pipelines, and reported literature trends. A multilayered 1D CNN tailored for biological Raman spectra, rather than depth for its own sake, achieved a mean accuracy of 0.8929 ± 0.0213, a sensitivity of 0.9086 ± 0.0321, a specificity of 0.8698 ± 0.0434, and an AUC of 0.9506 ± 0.0142 across 50 independent runs. The model’s best run achieved 92.9% accuracy and an AUC of 0.98. The Python implementation not only enables native integration with this and other AI/ML models, but also consolidates device control, preprocessing, visualization, archival, and inference into a single reproducible, extensible workflow independent of commercial software. We note that the present implementation emphasizes visual and user-guided auditability of different preprocessing steps and parameters rather than fully automated parameter optimization. Future work will focus on broader device compatibility, explainable feature importance, a dedicated spike-removal function, appropriate QC metrics, multisite, multicancer-type validation, cross-device robustness, and prospective intraoperative evaluation.

## Supplementary Material





## Data Availability

The data underlying this study are not publicly available at present. The biological Raman spectra are patient-derived and remain subject to ongoing research and institutional considerations. The manuscript and Supporting Information provide the methodological details, representative results, and processed summaries necessary to support the conclusions of this work.

## Notes

Supplementary video of RamanX Software Demo demonstrating the RamanX software workflow for data acquisition, preprocessing, visualization, and AI-based prediction (MP4)
Schematic representation of experimental setup; 3D printed light-deprived chamber; various auxiliary windows of RamanX; a picture of Tylenol’s packaging showing ingredients; comparison of this work’s results with previous data processing pipelines and commercial software; comparison of RamanNet with previous studies; comparison of RamanNet with popular ML models­(PDF)
