# Supplementary material for: An Integrated Raman Platform with Embedded AI for Intraoperative Real-Time Cancer Detection
Source: Anal Chem. 2026 Jul 10;98(30):22053–67. doi: 10.1021/acs.analchem.6c00260 (PMC13449223; doi:10.1021/acs.analchem.6c00260)
Supplement: Supplementary file 1 [file ac6c00260_si_003.pdf]

Supporting Information

An Integrated Raman Platform with Embedded AI for Intraoperative Real-Time Cancer Detection

Dinkar Regmi[1], Ya Zhang[1], Huaizhi Wang[1], Zheng Li[1], Jian Xu[1,*]

Author Information

[1]Division of Electrical and Computer Engineering, Louisiana State University, Baton Rouge, Louisiana 70803, USA

[*]Corresponding Author: jianxu1@lsu.edu

Table of Contents

Figure S1. Schematic representation of the experimental setup for data collection..........S-2
Figure S2. 3D Printed Light Deprivation Chamber..S-3
Figure S3. Simulation Mode in RamanX...S-4
Figure S4. Basic Settings Window...S-5
Figure S5. AI Selection Window..S-6
Figure S6. Filter View Window...S-7
Figure S7. Baseline View Window...S-8
Figure S8. Smooth & Normalized Window...S-9
Figure S9. Device Connection Window..S-10
Figure S10. Remove Background Window..S-11
Figure S11. Tylenol's Packaging Picture..S-12
Figure S12. Comparison of this work versus old work and commercial software.............S-13
Table S1. Comparison of RamanNet against prior studies...S-14
Table S2. Comparison of RamanNet with Conventional ML models...............................S-16
Supplementary Video Description...S-17

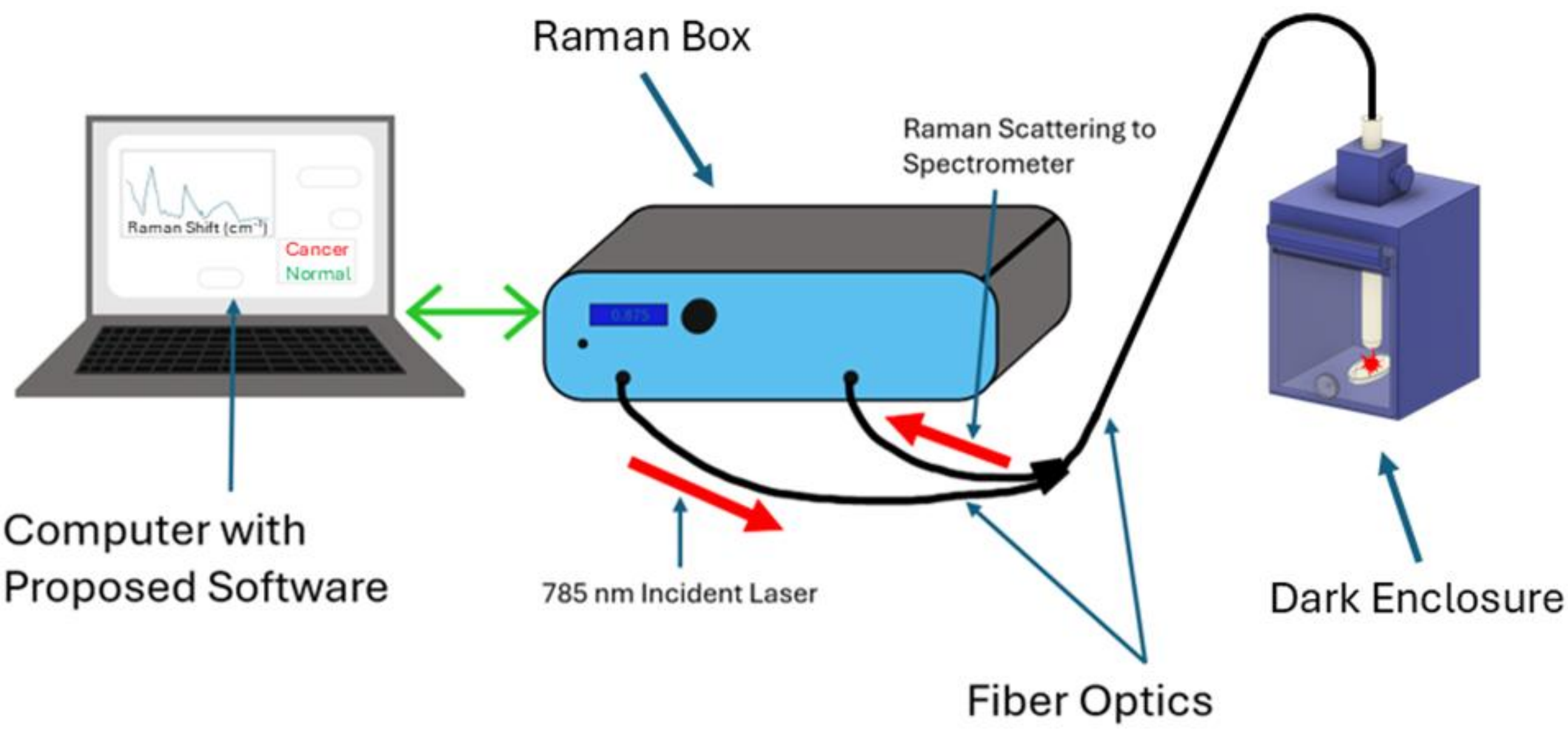

Figure S1: Schematic representation of the experimental setup for data collection of the non-biological sample.

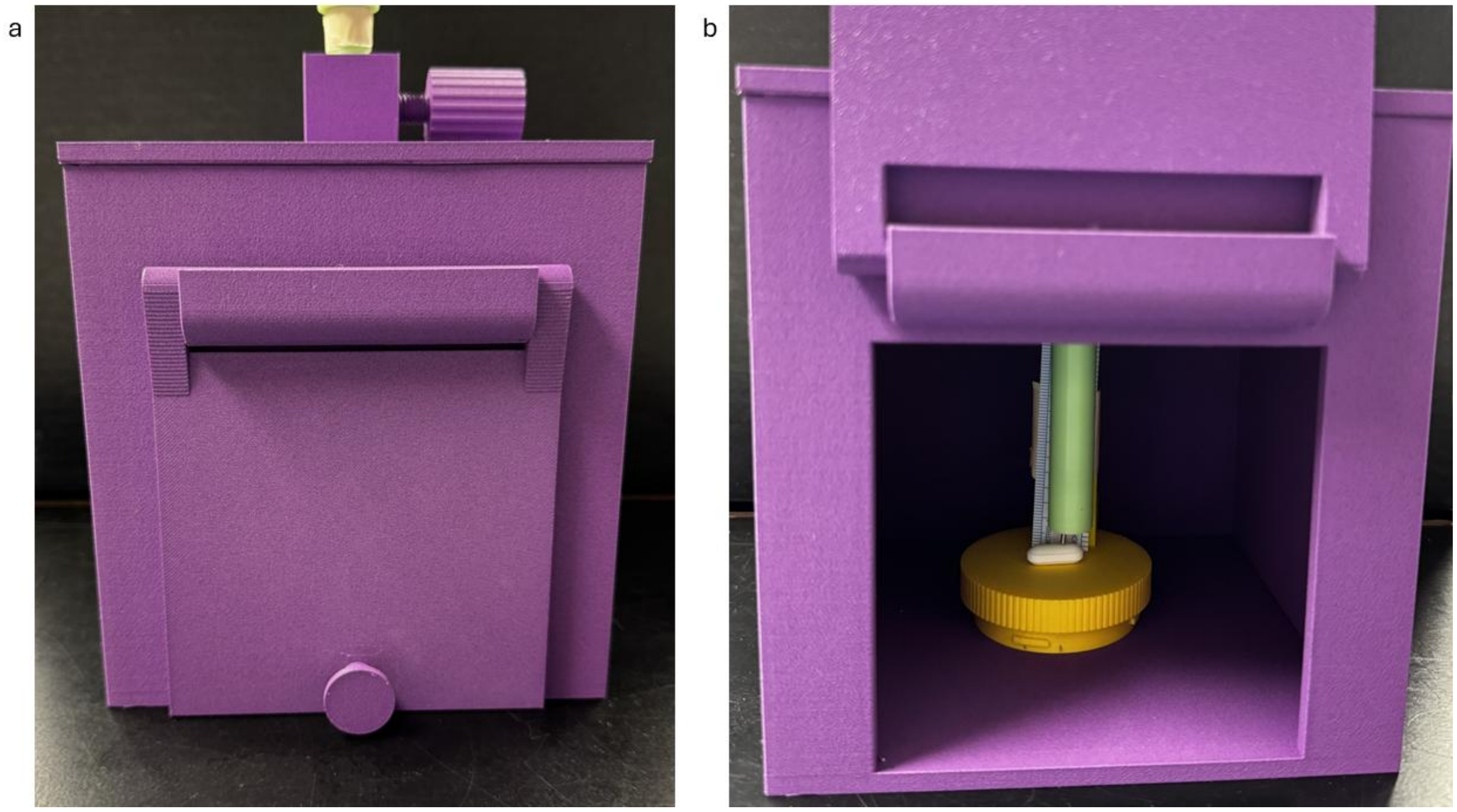

Figure S2: Light Deprivation Chamber with the door a) closed (during data collection), b) open.

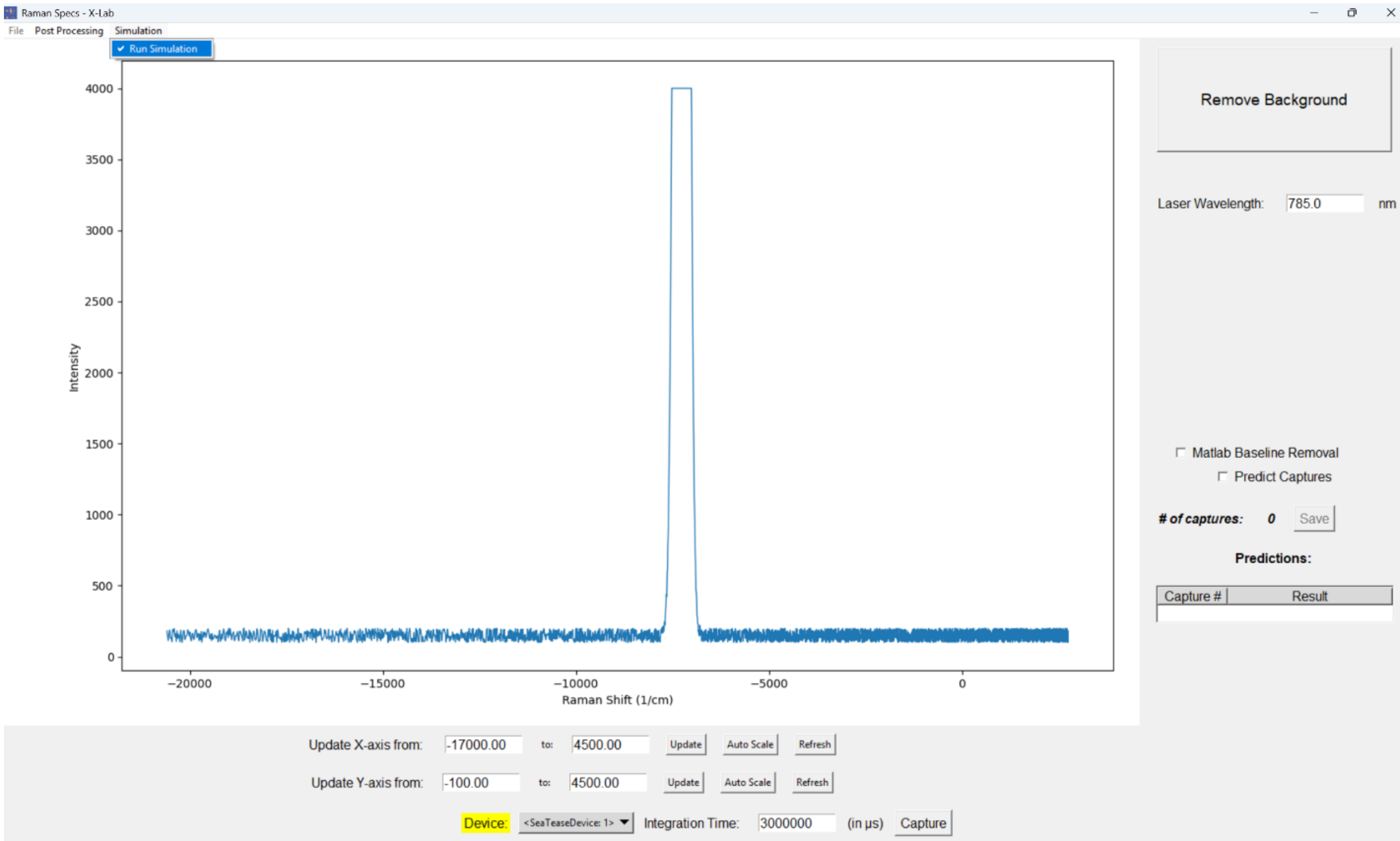

Figure S3: Simulation Mode using Seatease Python Module

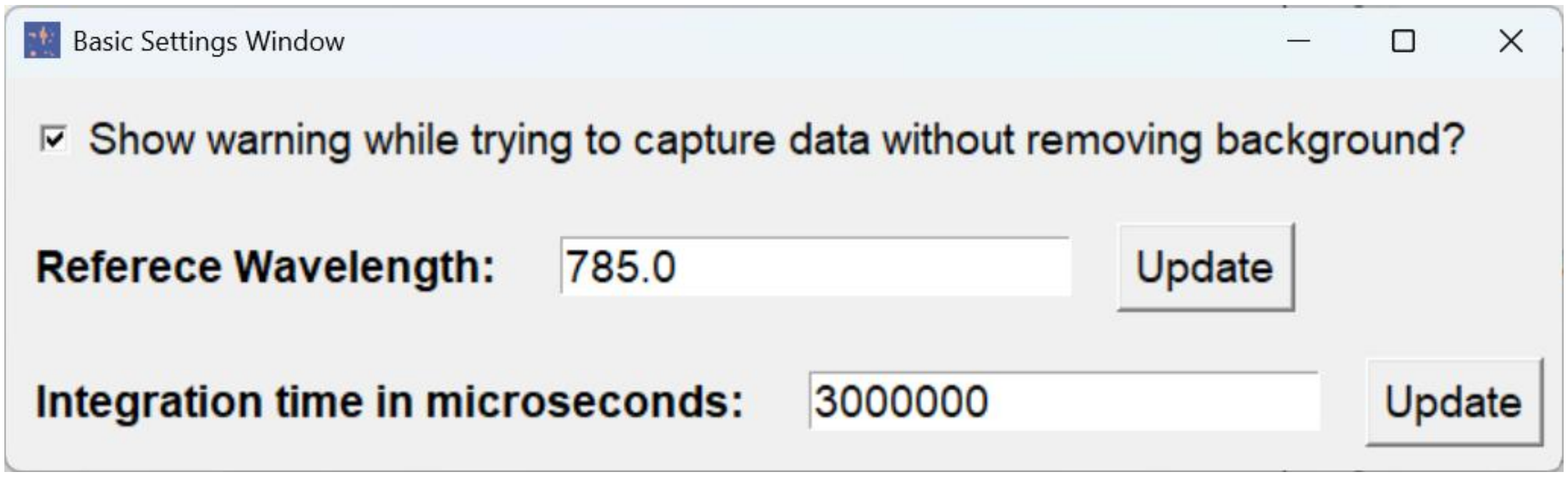

Figure S4: Basic Settings Window that allows to update reference wavelength value and the integration time.

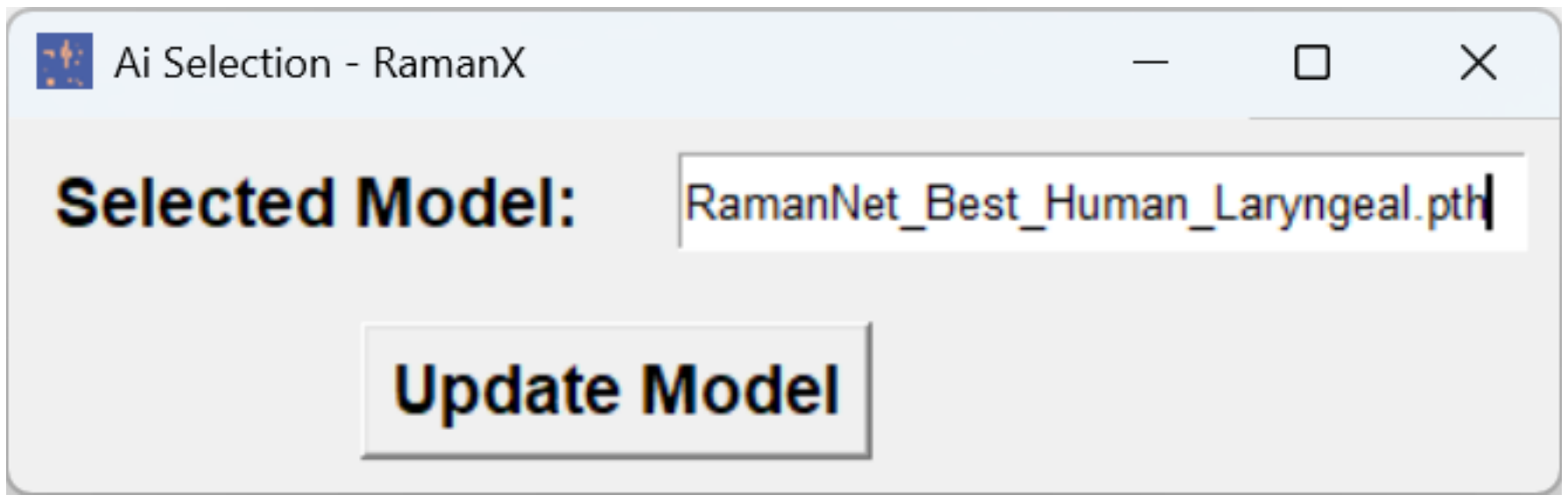

Figure S5: AI Model Selection Window. This window allows users to select an AI model from a list of available pre-trained models saved in a certain directory.

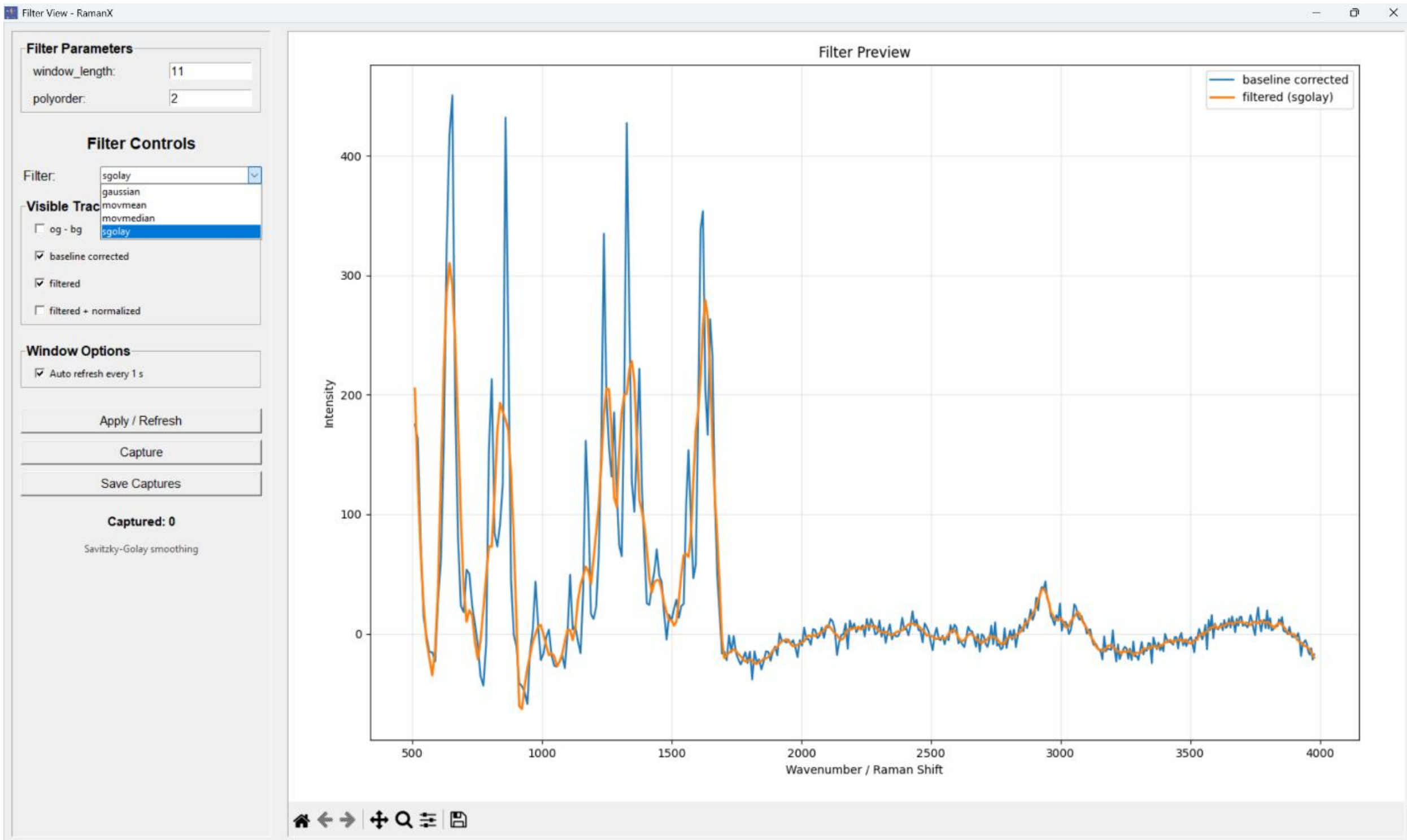

Figure S6: Filter View Window. Allows users to select from several smoothing filters and adjust related parameters.

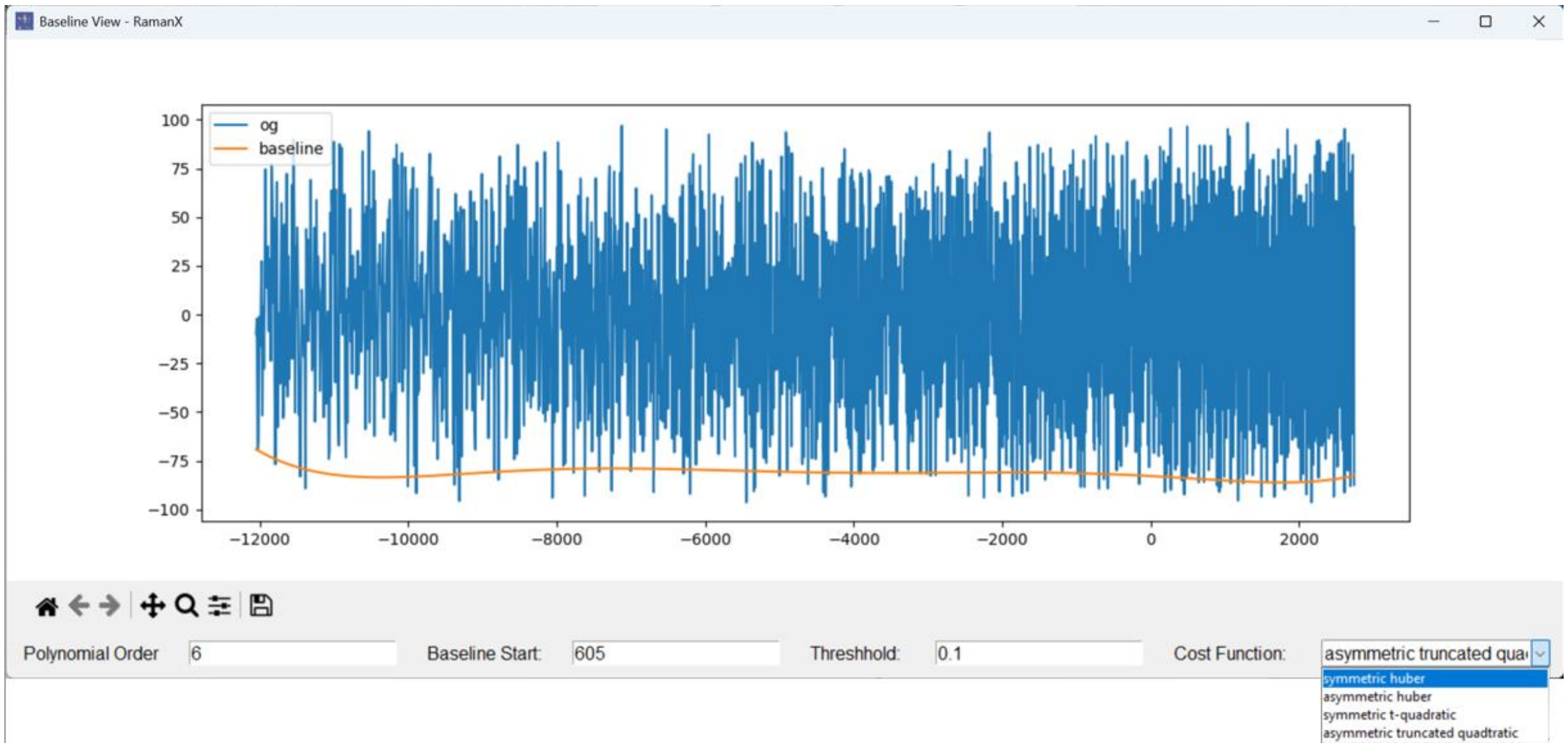

Figure S7: Baseline View Window. Allows users to see the real-time baseline and change variables being used to calculate the baseline including the cost function.

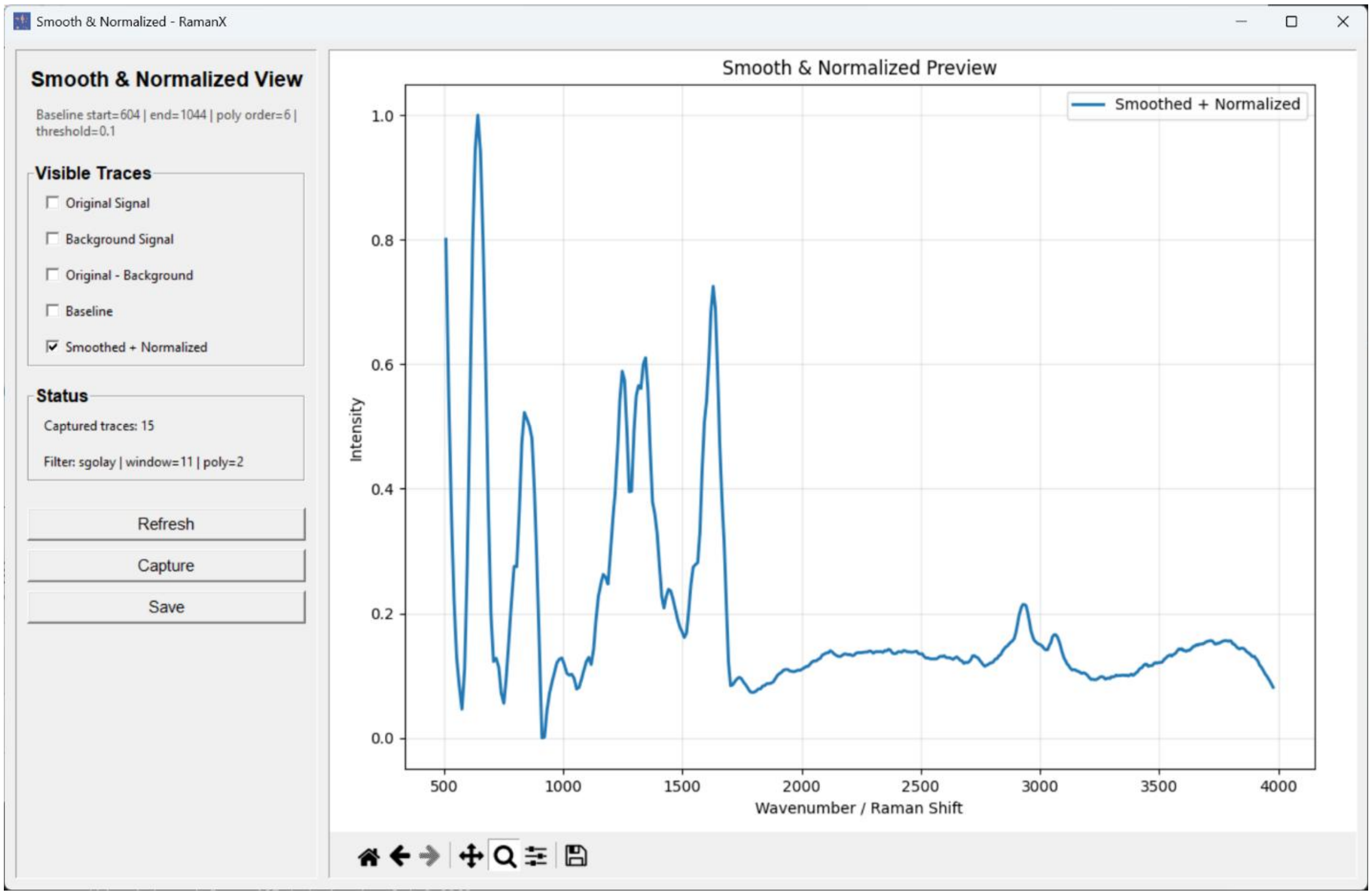

Figure S8: Smooth & Normalized View Window.

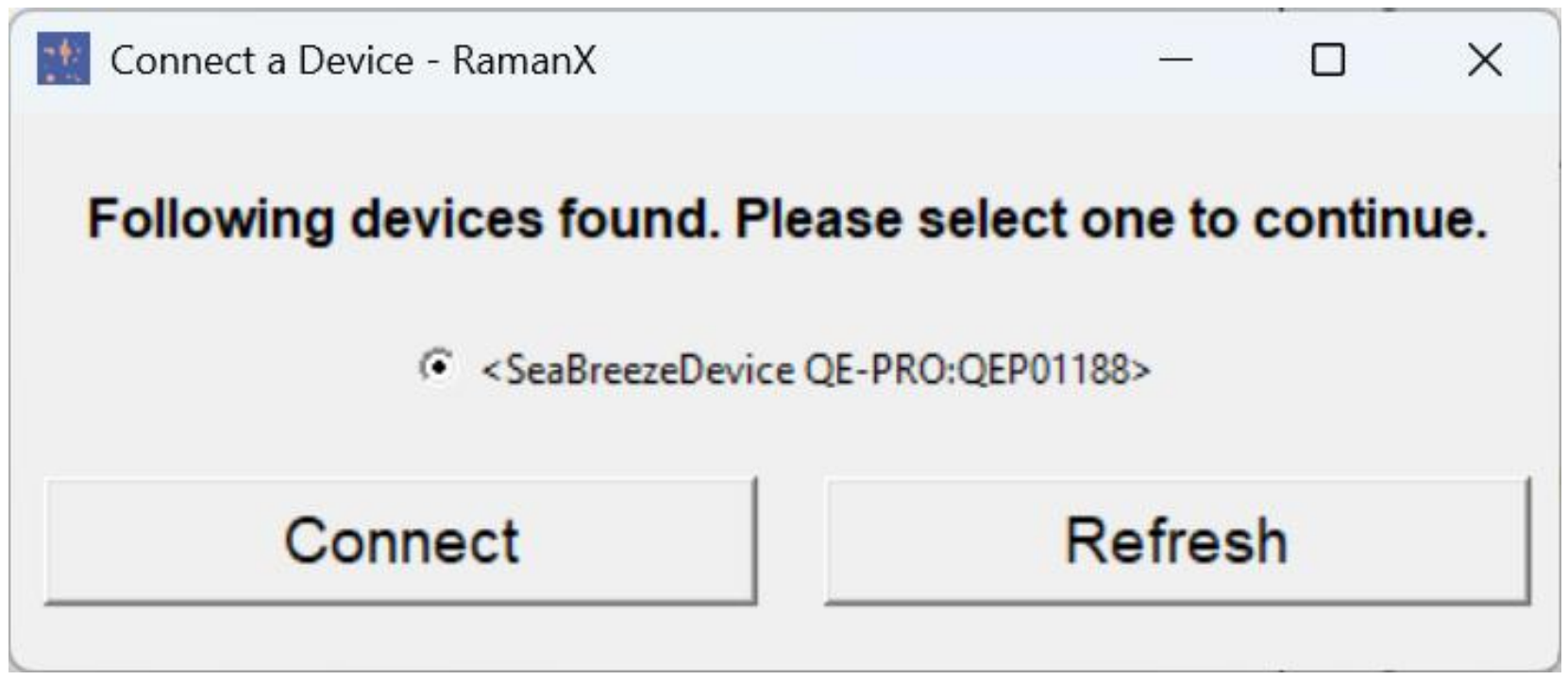

Figure S9: Device Connection Window. Scans for available devices and allows users to select a device.

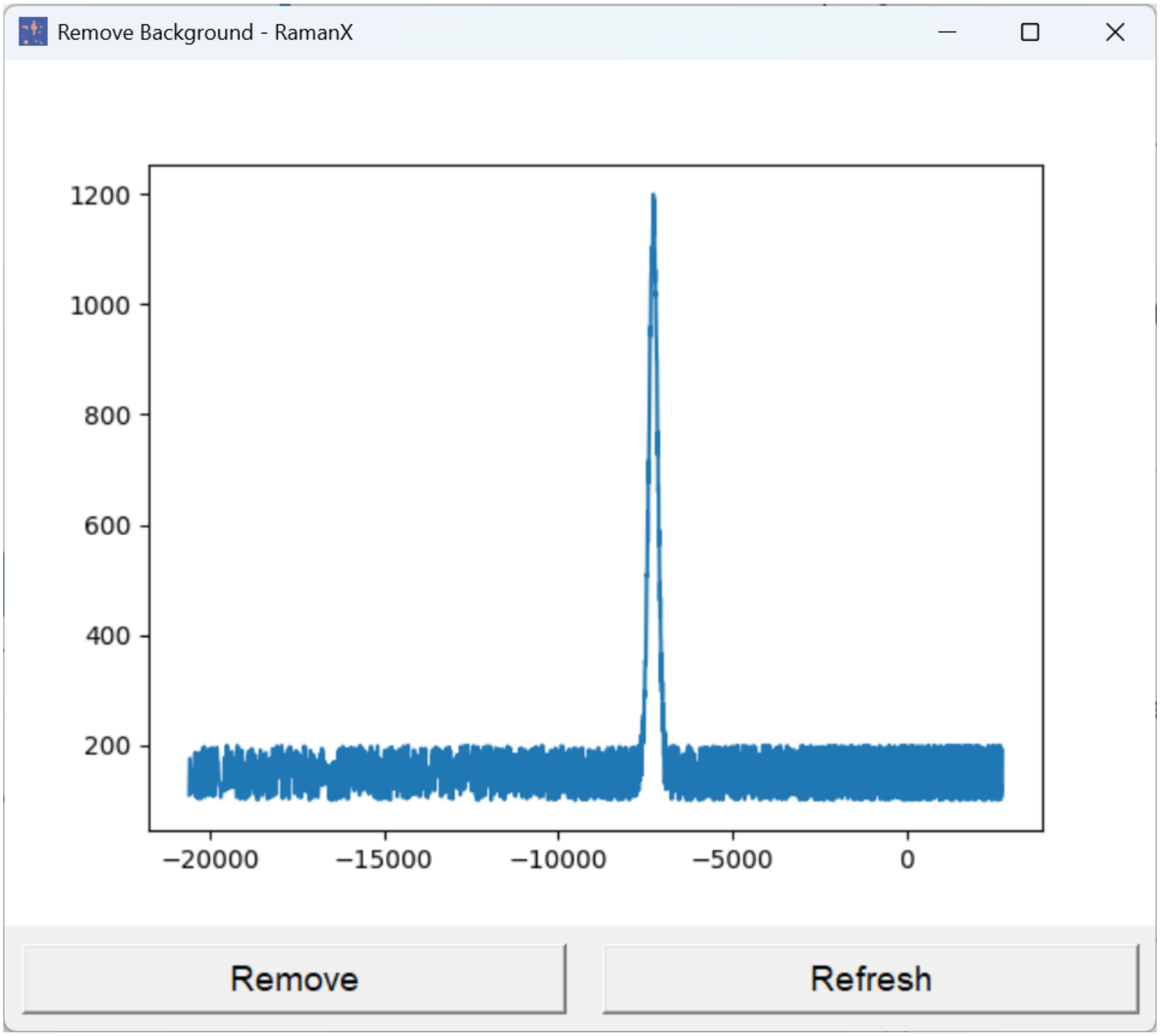

Figure S10: Remove Background Window.

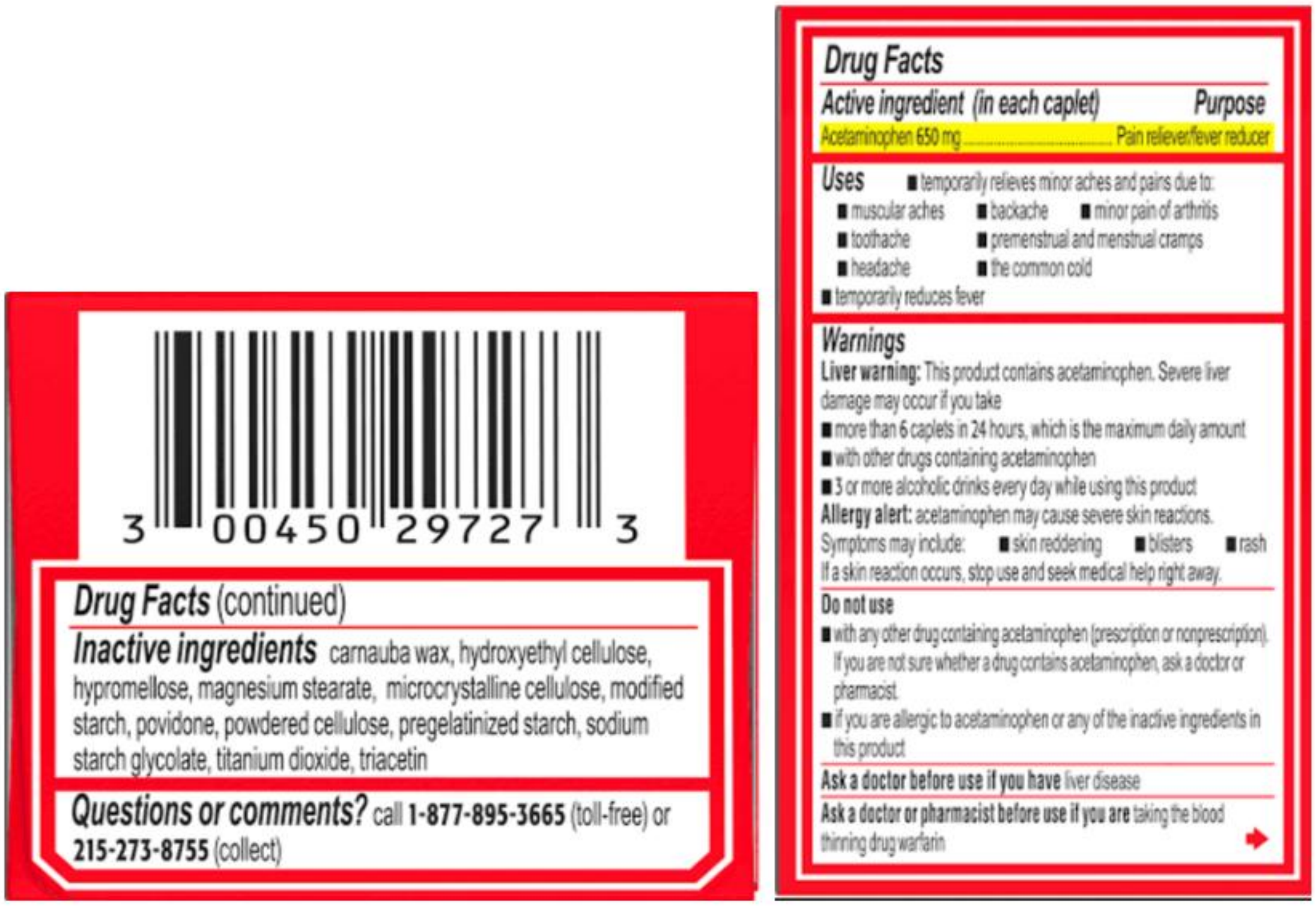

Figure S11: Picture on the packaging of the Tylenol tablets.

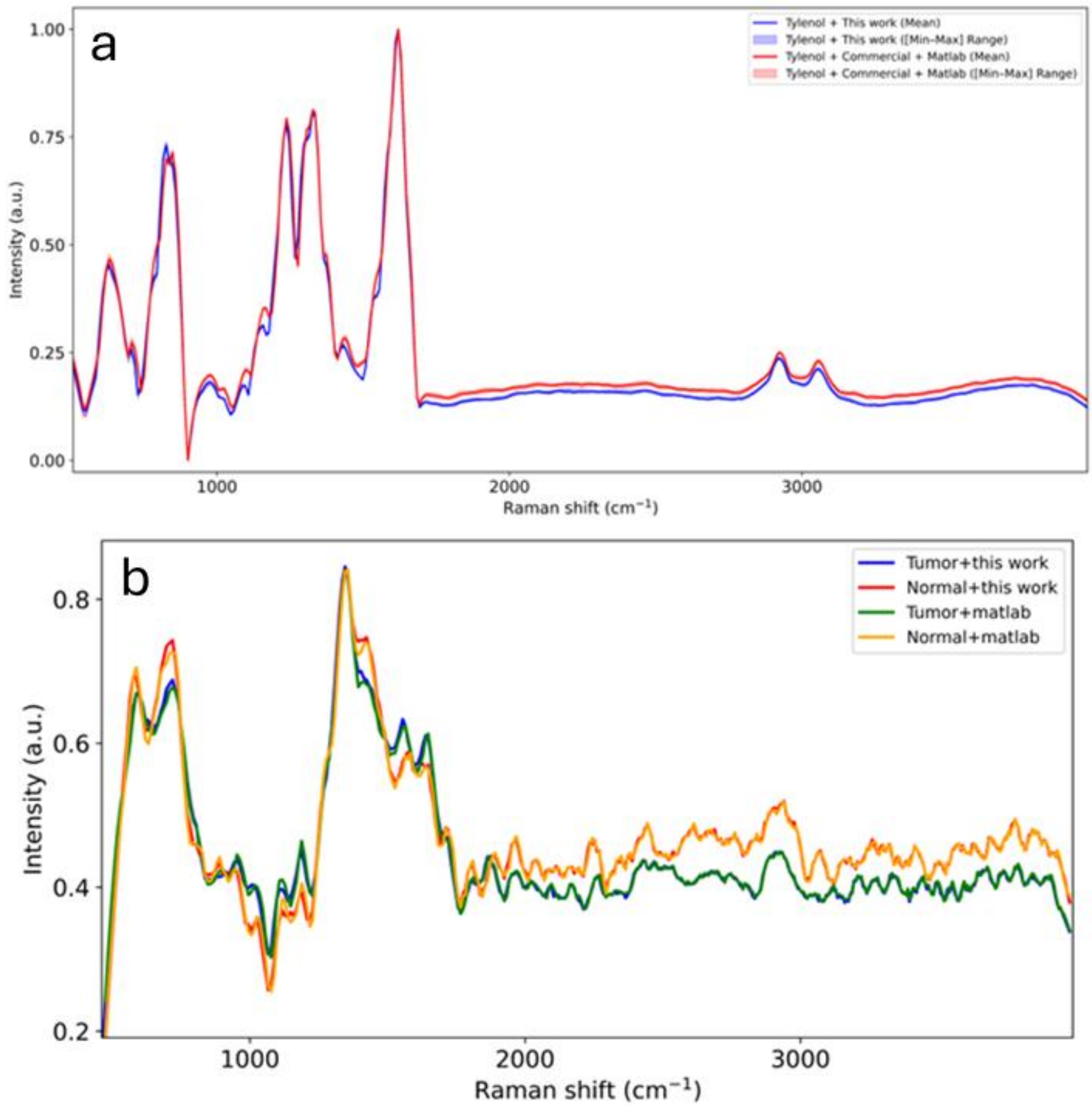

Figure S12: a) Normalized Raman spectra for Tylenol measured by our software (blue plot) vs commercial software and old MATLAB data processing pipelines (red plot), b) Average normalized Raman spectra of human laryngeal tissues obtained first by collecting data using commercial software then processing using new (this work) and old data processing pipelines. Blue and red plots respectively represent the average normalized Raman spectra for laryngeal cancer and normal tissues calculated by this work. Green and orange plots respectively represent the average normalized Raman spectra for laryngeal cancer and normal tissues calculated using old MATLAB data processing pipelines.

Table S1: Comparison of multilayered CNN (RamanNet) against prior studies.

Study	Number of Patients/Specimens	Number of Cancer Specimens	Number of Normal Specimens	Number of Cancer Spectra	Number of Normal Spectra	Total number of Spectra	Model	Accuracy (%)	Sensitivity (%)	Specificity (%)	AUC	Notes
This Study (best)	23	23	23	1320	892	2212	CNN	92.79	92.42	93.33	0.9784	tp/fp/tn/fn 122/6/84/10
This Study (average)	23	23	23	1320	892	2212	CNN	89.29 ± 2.13	90.86 ± 3.21	86.98 ± 4.34	0.9506 ± 0.0142	
LiCNN_Small (this study)	23	23	23	1320	892	2212	CNN	87.62 ± 2.14	89.77 ± 3.68	84.47 ± 4.71	0.9478 ± 0.0155	Lower Metrics + shallow CNN
Li et al., 2022[3]	10	10	10	500	207	707	CNN	96.1	95.2	96.9	0.98	Higher Metrics; but lower patient number + lower spectra + shallow CNN
Lin et al., 2016[32]	60	14	46	581	1321	1902	PLS-DA	91.1	93.3	90.1	0.968	Higher metrics than average; but lower number of cancer specimens + spectra + not CNN
Lin et al., 2012[38]	39	-	-	72	22	94	PCA-LDA	90.4	90.3	90.9	0.97	Higher metrics than average; Lower number of spectra + not CNN

Pujary et al., 2011[30]	10	10	10	60	48	108	PCA-based	-	75	75	-	Lower pateint number + lower spectra + not CNN + lower metrics
Teh et al., 2009[37]	16	30	20	117	70	187	RF	89.3	88	91.4	0.964	Lower pateint number + lower spectra + not CNN
Lau et al., 2005[36]	21	13	18	13	18	47	PCA/MANOVA + classifier	83	Carcinoma: 69.2	Carcinoma: 94.1	-	Lower cancer specimens + lower spectra + not CNN + lower metrics
Stone et al., 2009[35]	15	4	7	24	52	105	Linear discriminant analysis (stats)	82.9	Carcinoma:92<br>Normal: 83	Carcinoma:90<br>Normal: 94	-	Lower cancer specimens + lower spectra + not CNN + lower metrics
van Lanschot et al., 2023[39]	27	-	-	-	-	34	PCA-LDA	-	0.9	0.68	0.87	Lower spectra + not CNN + lower metrics

Table S2: Comparison of RamanNet with Conventional ML models with 50 runs

Model	Mean Accuracy	Accuracy Std.	Mean Sensitivity	Sensitivity Std.	Mean Specificity	Specificity Std.	Mean AUC	AUC Std.
RamanNet	0.8929	0.0213	0.9086	0.0321	0.8698	0.0434	0.9506	0.0142
RBFSVM	0.8889	0.0274	0.8795	0.0448	0.9027	0.0332	0.9323	0.0198
LinearSVM	0.8743	0.0254	0.873	0.0313	0.8762	0.0378	0.9461	0.0142
LogReg	0.8714	0.0252	0.8718	0.0302	0.8709	0.0383	0.9446	0.0154
RandomForest	0.8429	0.0229	0.9156	0.0251	0.7362	0.0452	0.9102	0.0176
PCA_LDA	0.8221	0.0248	0.8647	0.0319	0.7596	0.0439	0.891	0.0219
PLS_DA	0.6613	0.0164	0.998	0.004	0.1673	0.0396	0.9398	0.0154

Supplementary Video: Demonstration of RamanX Software Workflow

Supplementary Video RamanX Software Demo provides a tutorial-style demonstration of the RamanX platform developed in this study. The video shows the software workflow for Raman data acquisition and processing, including device connection, live spectral visualization, background removal, data saving, and optional AI-based real-time prediction.
